# IRE1/bZIP60-Mediated Unfolded Protein Response Plays Distinct Roles in Plant Immunity and Abiotic Stress Responses

**DOI:** 10.1371/journal.pone.0031944

**Published:** 2012-02-16

**Authors:** Adrian A. Moreno, M. Shahid Mukhtar, Francisca Blanco, Jon Lucas Boatwright, Ignacio Moreno, Melissa R. Jordan, Yani Chen, Federica Brandizzi, Xinnian Dong, Ariel Orellana, Karolina M. Pajerowska-Mukhtar

**Affiliations:** 1 FONDAP Center for Genome Regulation, Núcleo Milenio en Biotecnología Celular Vegetal, Centro de Biotecnología Vegetal, Facultad de Ciencias Biológicas, Universidad Andrés Bello, Santiago, Chile; 2 Department of Biology, University of Alabama at Birmingham, Birmingham, Alabama, United States of America; 3 Michigan State University–DOE Plant Research Laboratory and Department of Plant Biology, Michigan State University, East Lansing, Michigan, United States of America; 4 Department of Biology, Duke University, Durham, North Carolina, United States of America; Texas A&M University, United States of America

## Abstract

Endoplasmic reticulum (ER)-mediated protein secretion and quality control have been shown to play an important role in immune responses in both animals and plants. In mammals, the ER membrane-located IRE1 kinase/endoribonuclease, a key regulator of unfolded protein response (UPR), is required for plasma cell development to accommodate massive secretion of immunoglobulins. Plant cells can secrete the so-called pathogenesis-related (PR) proteins with antimicrobial activities upon pathogen challenge. However, whether IRE1 plays any role in plant immunity is not known. *Arabidopsis thaliana* has two copies of *IRE1*, *IRE1a* and *IRE1b*. Here, we show that both *IRE1a* and *IRE1b* are transcriptionally induced during chemically-induced ER stress, bacterial pathogen infection and treatment with the immune signal salicylic acid (SA). However, we found that IRE1a plays a predominant role in the secretion of PR proteins upon SA treatment. Consequently, the *ire1a* mutant plants show enhanced susceptibility to a bacterial pathogen and are deficient in establishing systemic acquired resistance (SAR), whereas *ire1b* is unaffected in these responses. We further demonstrate that the immune deficiency in *ire1a* is due to a defect in SA- and pathogen-triggered, IRE1-mediated cytoplasmic splicing of the bZIP60 mRNA, which encodes a transcription factor involved in the expression of UPR-responsive genes. Consistently, IRE1a is preferentially required for bZIP60 splicing upon pathogen infection, while IRE1b plays a major role in bZIP60 processing upon Tunicamycin (Tm)-induced stress. We also show that SA-dependent induction of UPR-responsive genes is altered in the *bzip60* mutant resulting in a moderate susceptibility to a bacterial pathogen. These results indicate that the IRE1/bZIP60 branch of UPR is a part of the plant response to pathogens for which the two Arabidopsis IRE1 isoforms play only partially overlapping roles and that IRE1 has both bZIP60-dependent and bZIP60-independent functions in plant immunity.

## Introduction

Plants and their pathogens are engaged in a constant, co-evolutionary battle for dominance. Unlike mammals, plants lack mobile phagocytic cells or somatic adaptive immune systems. However, they have evolved highly sophisticated innate immune systems to initiate effective defense responses [1], [2]. Plants recognize pathogens through membrane-associated and intracellular immune receptors. Upon pathogen recognition, plants trigger a robust disease resistance at the site of infection [3]. Stimulation of defense responses occurs not only locally but also in distal areas of the plant where the state of resistance is heightened, a phenomenon known as systemic acquired resistance (SAR) [4]. SAR confers immunity throughout the plant against a broad spectrum of pathogens. Activation of the SAR pathway involves an increase in the cellular concentration of the immune signal salicylic acid (SA), leading to dramatic induction of pathogenesis-related (*PR*) genes. In Arabidopsis, the SA signal is transduced through the central immune regulator NPR1 (Non-expressor of *PR* genes). Plants lacking functional NPR1 are impaired in their abilities to express *PR* genes and are almost completely defective in mounting SAR in response to pathogen infection [5], [6].

NPR1 is involved in the transcriptional changes of as many as ∼10% of genes in Arabidopsis upon treatment with SA [7], [8]. Among its direct transcriptional targets we found not only *PR* genes but also a large set of SAR- responsive endoplasmic reticulum (ER)-resident genes [9]. These ER-resident genes are up-regulated to ensure proper folding and secretion of the PR proteins, which are small polypeptides with antimicrobial activities, and to prevent accumulation of unfolded proteins [9]. Recently, it was proposed that a heat-shock like transcription factor TBF1 coordinately upregulates ER-resident genes upon biotic stimuli [10].

The cellular responses to unfolded proteins, collectively known as the unfolded protein response (UPR), have been studied extensively in yeast and humans [11]. The mammalian UPR signals through three ER-transmembrane proteins: IRE1, which resembles yeast IRE1/ERN11 (inositol-requiring and ER to nucleus signaling), ATF6 (activated transcription factor 6), and PERK (ER-resident PKR-like eIF2α kinase) [12]. These proteins represent three arms of the UPR. The UPR plays a fundamental role in maintaining cellular homeostasis and is therefore at the center of many normal physiological responses and pathologies [13]. In recent years, UPR has been shown to be involved in plasma cell differentiation in mammalian adaptive immunity as well as in innate immunity in invertebrates [14], [15], [16], [17]. However, it remains largely unknown whether UPR plays a role in plant immune responses and if it does, what are the molecular mechanisms involved in this process.

Genetic studies of the Arabidopsis *bip2* (luminal binding protein 2) mutant [9] suggest that the IRE1 branch of the UPR may play a role in plant immunity because the mutant of BiP, a known regulator of IRE1 in yeast [18], is defective in SAR. In yeast cells, engagement of BiP, an ER chaperone, modulates the activation and duration of UPR according to the magnitude of the cellular stress through its dynamic interaction with IRE1 [19]. Upon significant stress, a pool of IRE1 released from BiP can dimerize and cross-transphosphorylate to activate the IRE1 cytoplasmic endoribonuclease domains [20], [21]. The nuclease in turn cleaves two specific sites, defined by hairpins, in the mRNA encoding a basic leucine zipper (bZIP) transcription factor, mammalian *XBP-1* or yeast *HAC1*, in an unconventional cytoplasmic splicing event [22]. Consequently, the modified mRNA is produced that gives rise to an active transcription factor for the induction of ER-resident genes to enhance ER chaperone production [12].

The Arabidopsis genome encodes two IRE1s, IRE1a (At2g17520, formerly AtIre1-2) and IRE1b (At5g24360, formerly AtIre1-1) that share 41% amino acid identity, and the genes have largely overlapping expression patterns [23]. The kinase activation loop of IRE1a, but not IRE1b, is similar to the activation loop of mammalian IRE1 orthologs [24]. These findings suggest that IRE1a and IRE1b may have different physiological roles. Moreover, two recent reports show somewhat contrasting findings that describe either IRE1b alone [25] or both IRE1a and IRE1b [26] being required for the splicing of mRNA encoding bZIP60 (At1g42990), a basic leucine-zipper domain containing transcription factor, in response to heat and Tunicamycin (Tm; an inhibitor of *N*-linked glycosylation and a potent UPR inducer). The unspliced form of bZIP60 is translated into a protein containing cytoplasmic and transmembrane domains. However, under stress conditions, the processed bZIP60 mRNA is translated into a smaller protein that translocates to the nucleus and is required to regulate the expression of multiple ER-function related genes in a manner similar to HAC1 and XBP-1.

Here we show that plants lacking a functional IRE1 are hypersensitive to Tm. However, even though SA-dependent induction of UPR-responsive genes is affected in both *ire1a* and *ire1b* mutants, only *ire1a* has a significant effect on PR1 secretion in response to SA induction. Correspondingly, *ire1a* shows a pronounced disease susceptibility and deficient in SAR compared with *ire1b*, while *ire1a ire1b* plants show immune-related phenotypes of even further severity. Furthermore, we found that IRE1a and IRE1b are quantitatively required for Tm-, pathogen- and SA-induced bZIP60 splicing. Finally, we demonstrate that *bzip60* mutant is more sensitive to a virulent pathogen. Our results indicate that the IRE1/bZIP60 branch of the UPR signaling pathway plays distinct roles in plant immunity.

## Results

### Genes encoding IRE1 are involved in UPR induced by ER stresses

To investigate the role that UPR plays in response to stress, we employed a genetic approach. We obtained three independent mutants for *ire1a* (*ire1a-2*, *ire1a-3* and *ire1a-4*) and one mutant for *ire1b* (*ire1b-4*) (Figure S1) (see Materials and Methods). Additionally, we generated stable RNAi silencing lines for *IRE1b* in Col-0 and *ire1a-2* backgrounds, which show a severe depletion of both basal and induced IRE1b transcripts (Figure S2). Finally, we generated two independent double mutants (*ire1a-2 ire1b-4* and *ire1a-3 ire1b-4*) (see Materials and Methods). All of these mutants and transgenic plants were morphologically indistinguishable from wild-type under our growth conditions.

**Figure 1:**
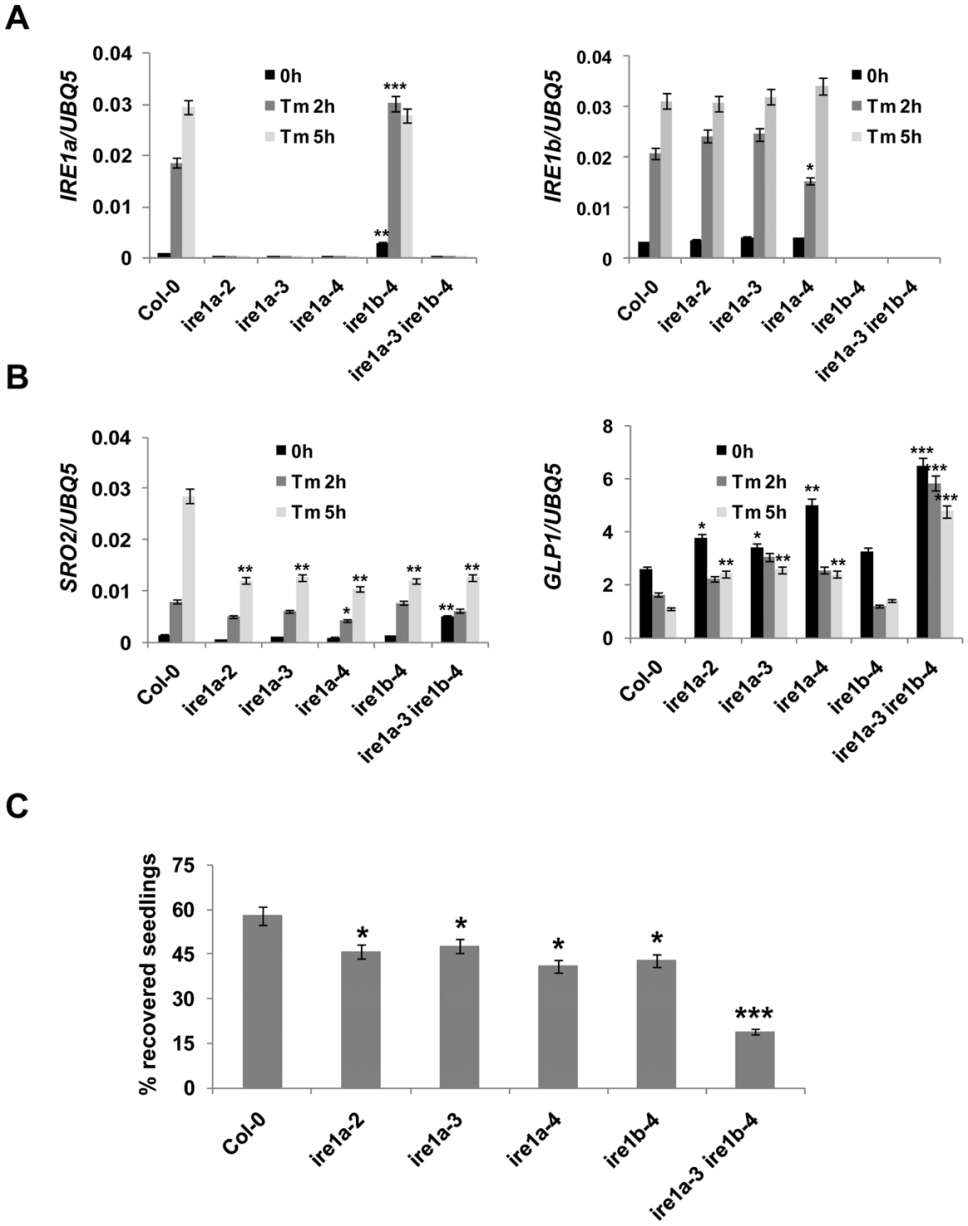
IRE1 is involved in abiotic stresses. **A,** The transcript accumulation of IRE1a and IRE1b and **B,** SRO2 and GLP1 in response to Tm treatment for 0, 2 and 5 hours in the listed genotypes measured by real-time RT-PCR. Induction of IRE1a, IRE1b, SRO2 and suppression of GLP1 can be visualized in the treated wild-type Col-0. IRE1a and IRE1b gene expression was analyzed to confirm the absence of mRNA in their respective T-DNA insertional mutants. Data represent the mean and SE of three technical replicates per treatment. Statistical analysis was performed using Student's *t*-test, *, *p*<0.05, **, *p*<0.01, ***, *p*≤0.001. Experiments with at least two independent biological replications demonstrate similar results. **C,** Abiotic-dependent UPR was induced in the wild-type and indicated mutant seedlings by growing them on MS medium containing 0.3 µg/mL Tm for three days. Percentage of recovery was plotted by calculating alive/dead seedlings recovered ten days post Tm treatment. Statistical analysis was performed using Student's *t*-test, *, *p*<0.05, ***, *p*≤0.001. Experiments were repeated at least three times with similar results.

To elucidate the function of IRE1 in UPR, we first examined the expression of both IRE1 genes upon treatment with Tm and observed a marked induction of IRE1a and IRE1b transcripts in wild-type Col-0 at 2 and 5 hours time points (Figure 1A). An experiment conducted in the *ire1* mutants showed that IRE1a and IRE1b are induced independently as the IRE1a expression in *ire1b-4* and the IRE1b transcript in *ire1a-2*, *ire1a-3* and *ire1a-4* mutants are comparable to Col-0.

During Tm-induced ER stress, as many as 259 genes are differentially expressed as a part of the UPR [27]. To examine the effects of *ire1* mutations on these UPR genes, we performed real-time PCR on two such ER stress markers genes, *SRO2* (Similar to RCD One 2) and *GLP1* (Germin-like protein 1) [27]. Consistent with the previous finding, we showed that Tm induces SRO2 expression, but represses GLP1 transcript levels in Col-0 plants (Figure 1B). The induction of SRO2 in *ire1a-2*, *ire1a-3*, *ire1a-4*, *ire1b-4* and *ire1a-3 ire1b-4* was significantly diminished, particularly at 5 hours post treatment. Importantly, we also observed a marked increase in the basal levels of SRO2 only in the *ire1a-3 ire1b-4* double knock-out mutant. This suggests that SRO2 is under transcriptional repression in an IRE1-dependent manner that is alleviated upon ER stress. Conversely, the basal transcript level of GLP1 was increased in all single *ire1a* and *ire1b* mutants and the effect was further pronounced in the *ire1a-3 ire1b-4* double mutant implying that both IRE1a and IRE1b are required in Tm-induced UPR.

To further illuminate the function of IRE1, we performed a recovery assay by growing *ire1* mutant seedlings in the presence of Tm for three days, followed by 10 days of growth on media without Tm. We were able to rescue over 60% wild-type seedlings (Figure 1C). In comparison with the wild-type and the untreated controls, *ire1a-2*, *ire1a-3*, *ire1a-4* and *ire1b-4* mutants exhibited noticeable growth retardation and chlorosis. This effect was further increased in the *ire1a-3 ire1b-4* double mutant with a significantly reduced recovery rate. The IRE1b RNAi lines showed phenotypic responses consistent with the insertional mutants (Figure S3). Taken together, these data suggest that both members of IRE1 additively function in Tm-induced ER stress.

### IRE1 plays an integral role in the secretion of PR1 in response to biotic stress

**Figure 2:**
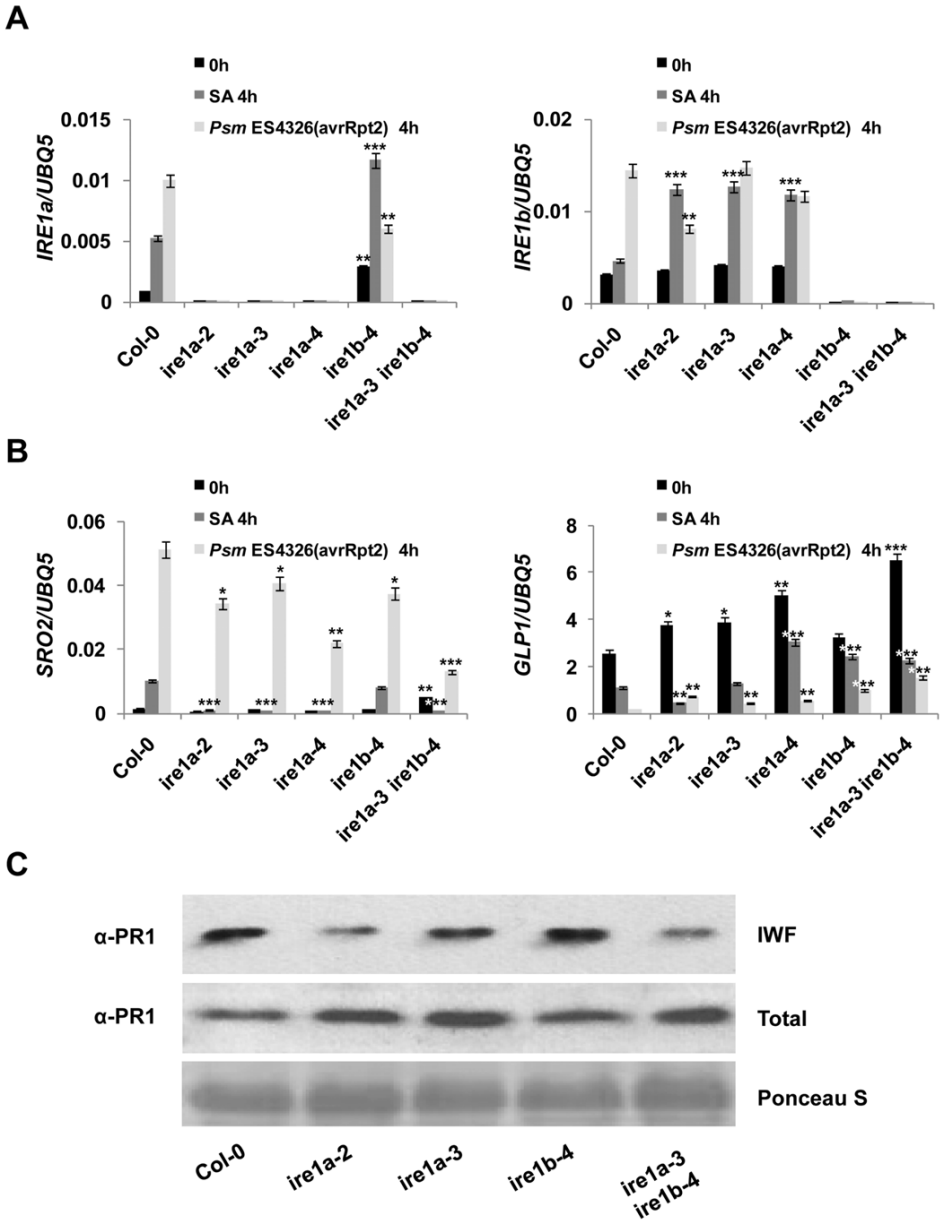
UPR-responsive genes and PR1 secretion is affected in *ire1* mutants. **A,** The expression of IRE1a and IRE1b and **B,** SRO2 and GLP1were quantified in response to SA and *Psm* ES4326(avrRpt2) for 4 hours in the indicated genotypes using real-time RT-PCR. Increased expression of IRE1a, IRE1b, SRO2 and reduced transcript of GLP1 can be observed in the treated wild-type Col-0. Data represent the mean and SE of three technical replicates per treatment. Statistical analysis was performed using Student's *t*-test, *, *p*<0.05, **, *p*<0.01, ***, *p*≤0.001. Experiments with at least two independent biological replications demonstrate similar results. **C,** PR1 protein accumulation in the *ire1* mutants was compared with wild-type. Intercellular wash fluid (IWF) was collected from 20 leaves derived from 10 plants per indicated genotype treated with SA for 16 hours. Total protein was extracted from five leaves derived from three plants per indicated genotype treated with SA for 16 hours. Accumulation of PR1 was detected by Western blotting with anti-PR1 antibody in IWF and total leaf extract from the indicated genotypes. Ponceau S stain verifies equal loading. Experiments were repeated at least four times with similar results.

SA, a major phytohormone, regulates over 2000 genes in Arabidopsis [8]. We investigated the induction of IRE1a and IRE1b upon SA treatment and pathogen infection with *Pseudomonas syringae* pv. *maculicola* strain ES4326 expressing the avrRpt2 type III effector, hereafter referred to as *Psm* ES4326(avrRpt2). Expression levels of both IRE1a and IRE1b were considerably increased upon both SA and *Psm* ES4326(avrRpt2) application at 4 hours in wild-type Col-0 plants (Figure 2A). Moreover, both SA and pathogen markedly induced IRE1a expression in *ire1b-4* and IRE1b transcript in *ire1a-2*, *ire1a-3* and *ire1a-4* mutants. We then tested the two key genes encoding UPR-responsive markers in the *ire1* mutants in response to SA and *Psm* ES4326(avrRpt2) induction (Figure 2B). The 0 h samples were shared between the Tm, SA and *Psm* ES4326(avrRpt2) treatments to better compare the results obtained from biotic and abiotic stresses. We observed the suppression of SRO2 transcripts in single *ire1a-2*, *ire1a-3*, *ire1a-4*, *ire4b* and double *ire1a-3ire1b-4* mutants upon SA treatment as well as *Psm* ES4326(avrRpt2) infection. We detected an increase in GLP1 basal transcript level in all single *ire1a* and *ire1b* mutants and this effect was further enhanced in the *ire1a-3 ire1b-4* double mutant.

These data suggested that *ire1a* and *ire1b* have a defect in SA- and pathogen-induced transcription of genes encoding the ER machinery, and this defect might further translate into impairment in secretion. To test this hypothesis, we examined the secretion of pathogenesis-related 1 (PR1) protein, a hallmark of inducible immune response in Arabidopsis. We collected intercellular wash fluid (IWF) from the leaves of wild-type, *ire1a-2*, *ire1a-3*, *ire1b-4* and *ire1a-3 ire1b-4* mutants that were treated with SA for 16 hours. We observed a marked reduction of secreted PR1 accumulation in *ire1a-2* and *ire1a-3* mutants, but not in the *ire1b-4*, when compared to the wild-type (Figure 2C). PR1 secretion was further reduced in the *ire1a-3 ire1b-4* double mutants. Examination of total PR1 levels further supported our conclusion that IRE1 are required for PR1 secretion, but not protein expression (Figure 2C). This result was further validated in IRE1b RNAi lines in *ire1a-2* background (Figure S4). These data demonstrate that IRE1s, especially IRE1a, play an important role in plant defense by controlling the secretion of antimicrobial proteins.

### Plants lacking functional IRE1 genes are impaired in establishing SAR

**Figure 3:**
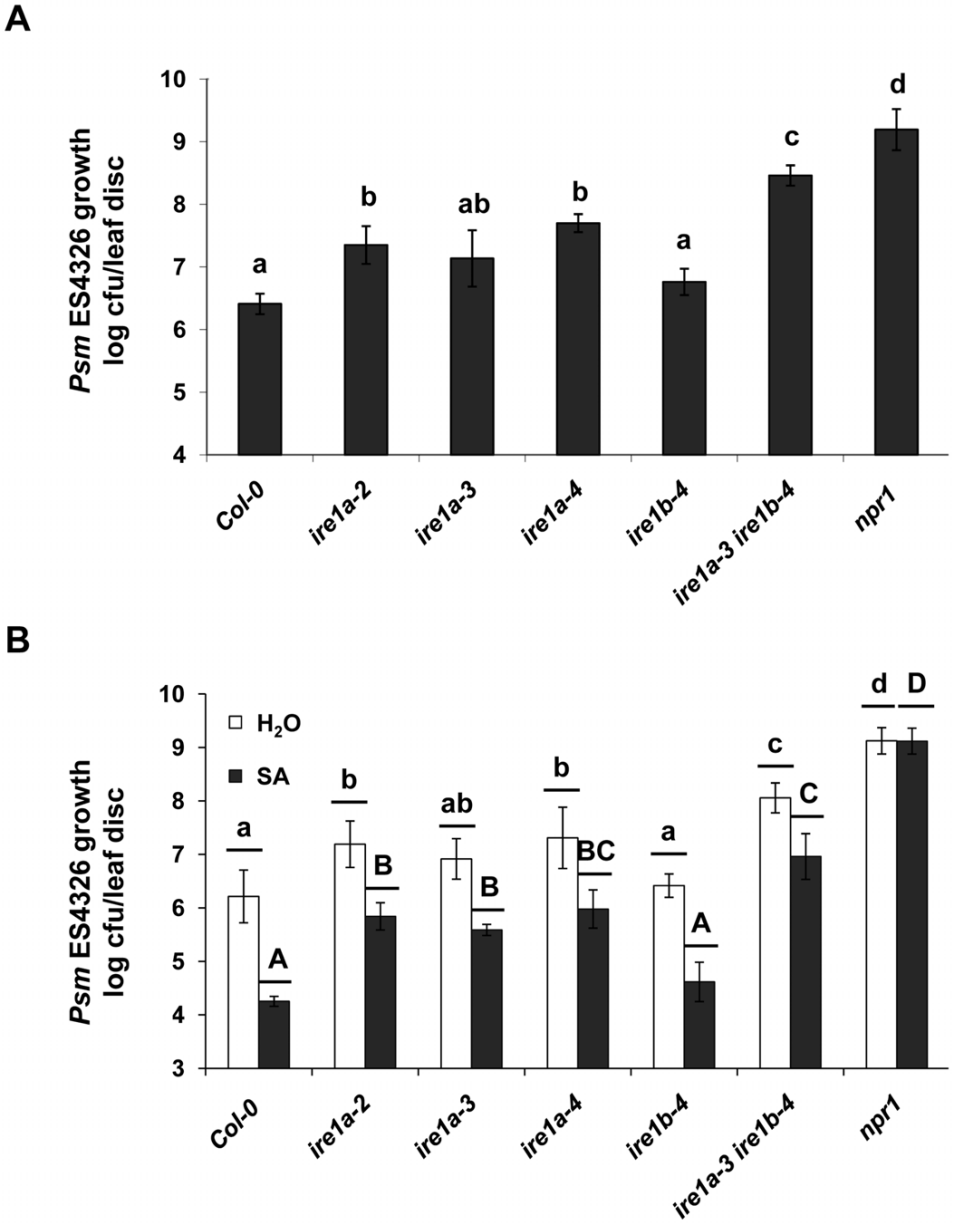
IRE1 is required to mount effective systemic acquired resistance. **A,** Bacterial growth (colony forming unit – cfu/leaf disc, expressed on a log scale) of leaves of the indicated genotypes infected with *Psm* ES4326 (OD = 0.0002). Bacterial growth was assessed at 3 dpi. Hypersusceptible *npr1* mutant was used as control. Error bars: 95% confidence interval of the mean (*n* = 8). Bars connected by the same letter did not differ from each other at *p*<0.05 (Tukey's HSD tests). **B,** Chemical SAR was established by treating indicated genotypes with 1 mM SA, while uninduced plants were sprayed with water 16 hours prior to *Psm* ES4326 (OD = 0.001). Bacterial growth was monitored 3 days post infection. Hypersusceptible *npr1* mutant was used as control. Error bars represent 95% confidence interval of the mean (*n* = 8). Bars within a class connected by the same letter (lowercase for water treatment; uppercase for SA treatment) did not differ from each other at *p*<0.05 (Tukey's HSD tests). All the experiments were performed at least three times with similar results.

Since the SA-dependent regulation of UPR genes is affected and the secretion of PR1 is diminished in *ire1* mutants, we reasoned that loss-of-function of IRE1 might result in compromised disease resistance responses. Thus, we performed an enhanced disease susceptibility (EDS) test in wild-type, various single and double *ire1* knock-out plants and *npr1* mutants using a low dose of virulent bacterial pathogen *Psm* ES4326 (OD = 0.0002). At this dose of inoculant, the immune-deficient *npr1* mutant showed a 1000-fold more bacterial growth than the wild-type (Figure 3A). Under the same conditions, we observed a 10-fold increase in bacterial population in *ire1a-2*, *ire1a-3*, and *ire1a-4* compared to Col-0 plants. In contrast, no EDS phenotype was observed in *ire1b-4* plants. However, plants lacking both *IRE1* genes (*ire1a-3 ire1b-4* and IRE1b RNAi lines) exhibited up to 100 fold higher bacterial growth compared to wild-type (Figures 3A, S5).

We next tested whether *IRE1* genes are also required to establish effective SAR by spraying plants with SA, followed by infection with a higher dose of *Psm* ES4326 (OD = 0.001) 16 hours later. In wild-type plants a 100-fold reduction in bacterial population was observed whereas in *npr1* mutant no SAR was detected (Figure 3B). In comparison, SA-treated *ire1a-2*, *ire1a-3* and *ire1a-4* had an approximately 100-fold higher bacterial population compared to similarly treated wild-type plants. Interestingly, loss-of-function of IRE1b was not defective in establishing SAR. However, SA-treated *ire1a-3 ire1b-4* supported 1000 times more bacterial growth compared to SA-treated wild-type. We concluded that *ire1a-3 ire1b-4* failed to induce effective SAR, most likely due to mis-regulation of ER-resident genes, and subsequently a defect in secretion of PR1 into the apoplast. Similar results were also obtained using the IRE1b RNAi lines in Col-0 and *ire1a-2* backgrounds (Figures S5, S6).

### Quantitative requirement of functional IRE1a and IRE1b in bZIP60 mRNA processing upon abiotic stresses

**Figure 4:**
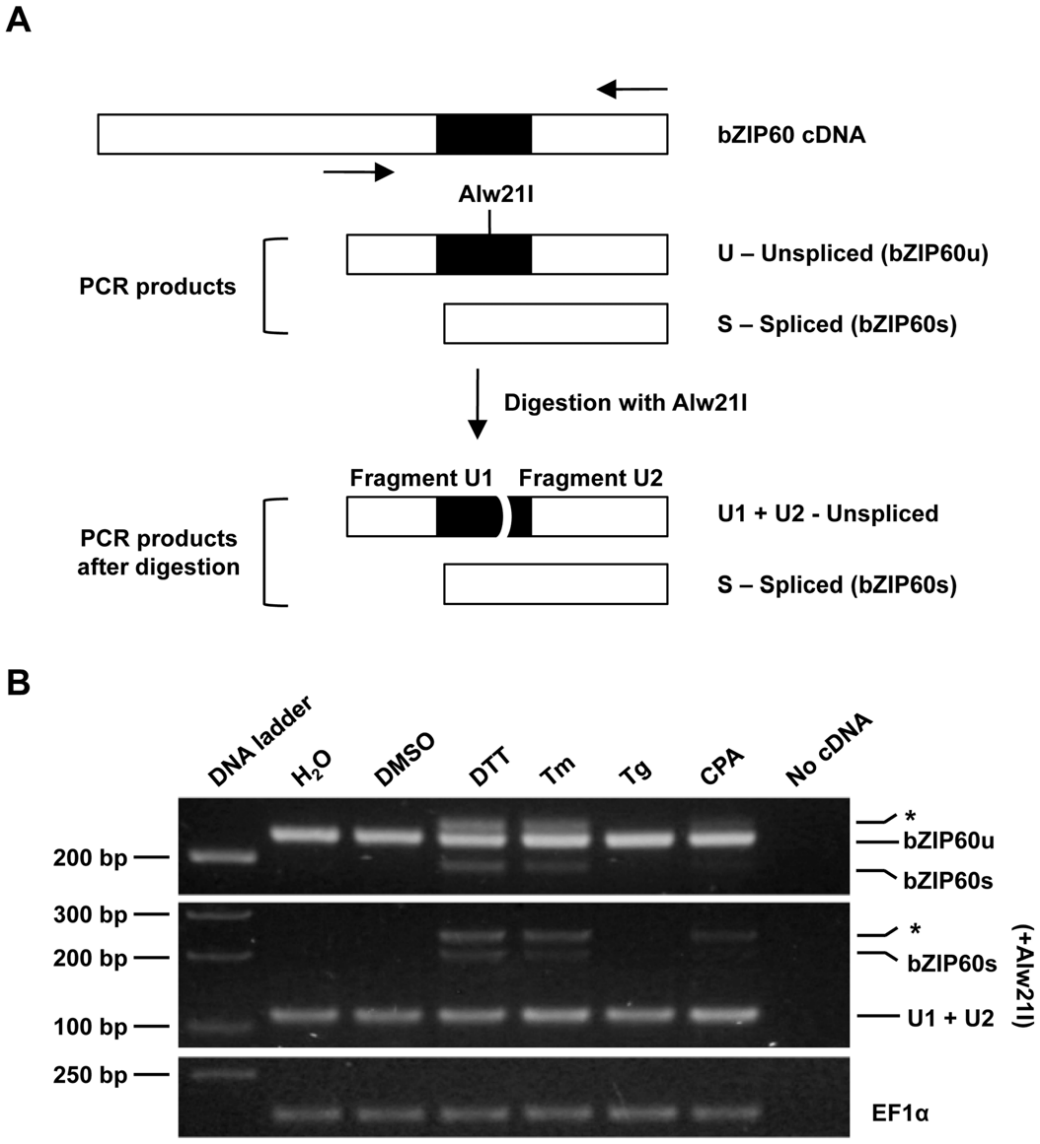
bZIP60 mRNA splicing is stimulated by chemicals that trigger the UPR. **A,** Schematic representation of two approaches used to detect the bZIP60 mRNA spliced forms. Primers sets flanking the putative splicing regions (solid arrows) are indicated (Top) to amplify bZIP60u and bZIP60s forms using RT-PCR. Alternative, RT-PCR products are subsequently digested using Alw21I restriction enzyme (Bottom). The latter approach will highlight the length differences between bZIP60u and bZIP60s since the Alw21I restriction site is present in bZIP60u and absent in bZIP60s. bZIP60u and bZIP60s PCR products upon digestion are shown. **B,** Processing of bZIP60 mRNA was analyzed by gel electrophoresis in agarose (3.5% p/v). RT-PCR products (Top) or RT-PCR products digested with Alw21I (bottom) were obtained from RNA samples of Arabidopsis seedlings (6-day-old) treated for 2 hours with several chemicals that trigger the UPR (Tm 5 µg/mL; DTT 5 mM; CPA 100 µg/mL; Thapsigargin 500 nM). DMSO and water-treated samples served as mock controls for chemicals. Asterisk indicates a hybrid band formed by the bZIP60u and bZIP60s PCR products. Such hybrid band has been also observed and documented in RT-PCR analysis of XBP-1 processing [56]. Elongation factor 1 alpha (EF1α) expression served as a control. All the experiments were performed at least three times with similar results.

Activation of IRE1 in yeast and humans leads to cytoplasmic splicing of HAC1 and XBP-1 mRNAs, respectively, and induction of downstream UPR genes. The candidate for this IRE1-regulated transcription factor in Arabidopsis, bZIP60, was identified through a search for hairpins similar to those required for HAC1 and XBP-1 mRNA splicing [25], [26] (Figure S7). However, it was not known whether bZIP60 is indeed a target of IRE1 nuclease activity in response to biotic stresses such as pathogen infection. Moreover, the quantitative contribution of each IRE1 homolog in response to biotic and abiotic stresses was not clear. Treating plants with DTT and Tm, two known inducers of UPR, we observed the appearance of an additional bZIP60 amplicon smaller in size (bZIP60s) in the RT-PCR experiment (Figure 4). Sequence analysis confirmed that bZIP60s corresponds to a processed form of bZIP60, lacking 23 nucleotides, compared to the unspliced bZIP60 (bZIP60u) (Figure S8). We also found that the ER calcium pump blocker cyclopiazonic acid (CPA) can induce the processing of bZIP60. In addition, we used thapsigargin, another blocker of calcium ATPase pumps, and showed that it has no effect on bZIP60 splicing.

**Figure 5:**
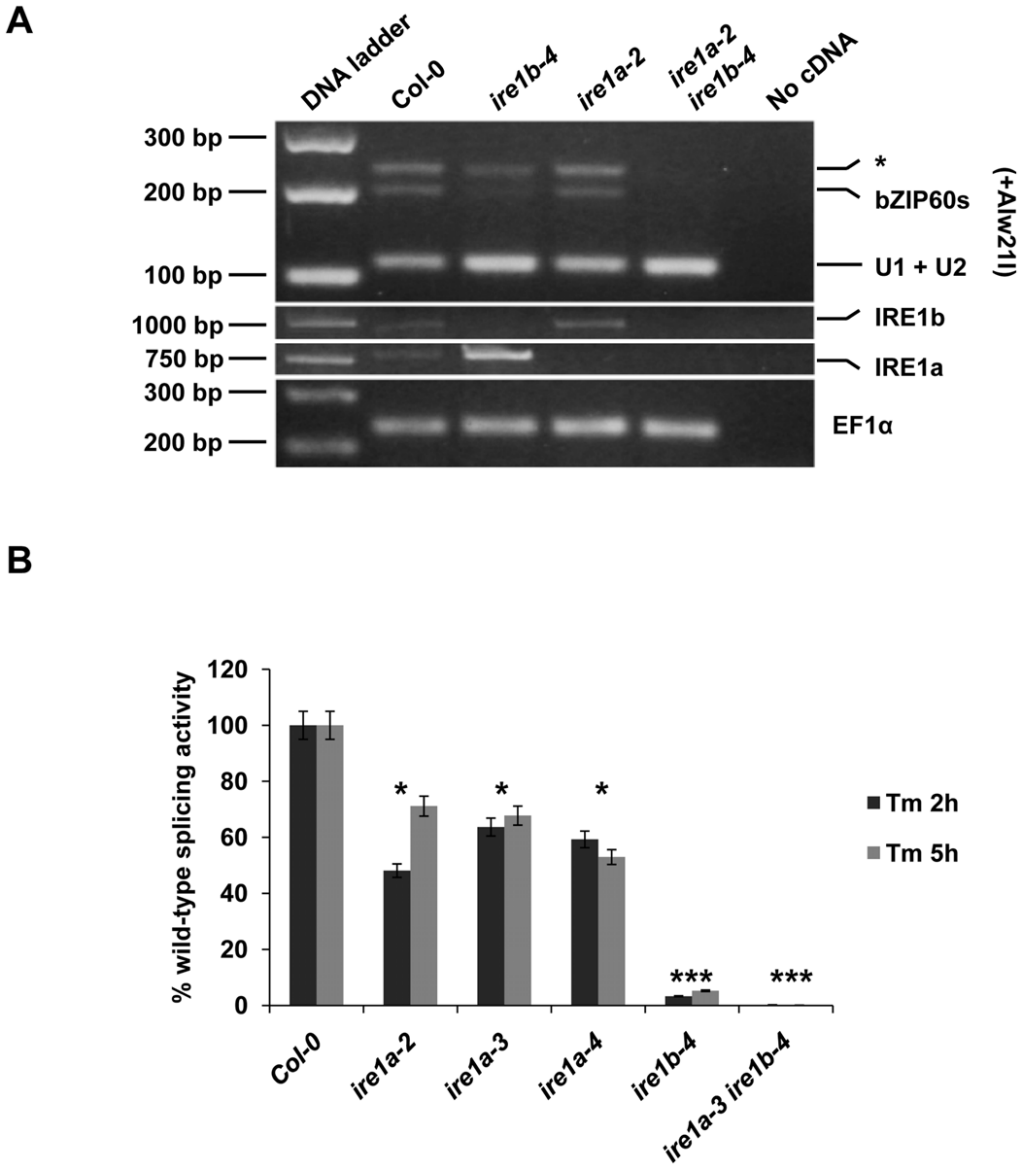
T-DNA insertions in both IRE1 genes affectbZIP60 processing under ER stress conditions. **A,** RT-PCR products derived from bZIP60 mRNA were digested with Alw21I and resolved by gel electrophoresis in agarose (3.5% p/v). RNA samples were obtained from wild-type or *ire1b-4*, *ire1a-2* and *ire1a-2 ire1b-4* mutant seedlings (6-day-old) treated with Tm for 2 hours. IRE1b and IRE1a gene expression was analyzed to confirm the absence of mRNA in their respective T-DNA insertional mutants. Elongation factor 1 alpha (EF1α) gene expression served as a control. **B,** Quantitative measurement of bZIP60 splicing activity. cDNA was made from the leaf tissue of 3-week-old plants of the indicated genotypes, untreated or infiltrated with 0.5 µg/mL Tm for 2 hours and 5 hours. Ratios of fold induction of spliced and unspliced bZIP60 are plotted, while setting ratio of Col-0 as 100%. Statistical analysis was performed using Student's *t*-test, *, *p*<0.05, ***, *p*≤0.001. All the experiments were performed at least three times with similar results.

Given the position and structure of the processing site in the bZIP60 mRNA, we and others proposed that bZIP60s is generated through unconventional splicing mediated by IRE1 (Figure S7) [25], [26]. This hypothesis is clearly supported by the significantly reduced and abolished bZIP60 mRNA processing upon Tm treatment in the *ire1b-4* single and the *ire1a-2 ire1b-4* double mutant, respectively (Figure 5A). Interestingly, this Tm-induced bZIP60 splicing seems to predominantly require IRE1b as the *ire1a-2* mutant showed a near-wild-type level of bZIP60 processing.

Recently, two reports describe the requirement of only IRE1b [25] or both IRE1a and IRE1b [26] for bZIP60 processing during heat- and/or Tm-induced UPR. These studies were based on the presence or absence of the bZIP60s amplicon. To gain deeper insight into the requirement of IRE1 proteins in bZIP60 processing, we developed a quantitative transcript measurement assay using real-time quantitative RT-PCR that can distinguish between the bZIP60u and bZIP60s forms (Figure 5B) (see Materials and Methods). A similar method was recently employed to demonstrate the quantitative changes of IRE1-dependent XBP-1 processing in *Caenorhabditis elegans* upon infection with *Pseudomonas aeruginosa* [16] and in a human acute monocytic leukemia cell line [28]. We showed that all *ire1a* mutants maintain 50–65% of the bZIP60 splicing activity, while *ire1b* reduces bZIP60 processing by 95%. bZIP60 splicing was completely abolished in *ire1a-3 ire1b-4*. This is consistent with the results from the regular RT-PCR analysis and with another recently published report [25]. Our data was further supported by the reduction of bZIP60 splicing activities in IRE1b RNAi lines (Figure S9).

We also tested whether the exposure of Arabidopsis plants to other abiotic stresses had an effect on bZIP60 processing. We showed that heat can promote bZIP60 mRNA splicing, but salt, cold and osmotic stresses failed to induce bZIP60 processing (Figure S10).

### Pathogen infection- and SA-dependent bZIP60 processing preferentially requires IRE1a

**Figure 6:**
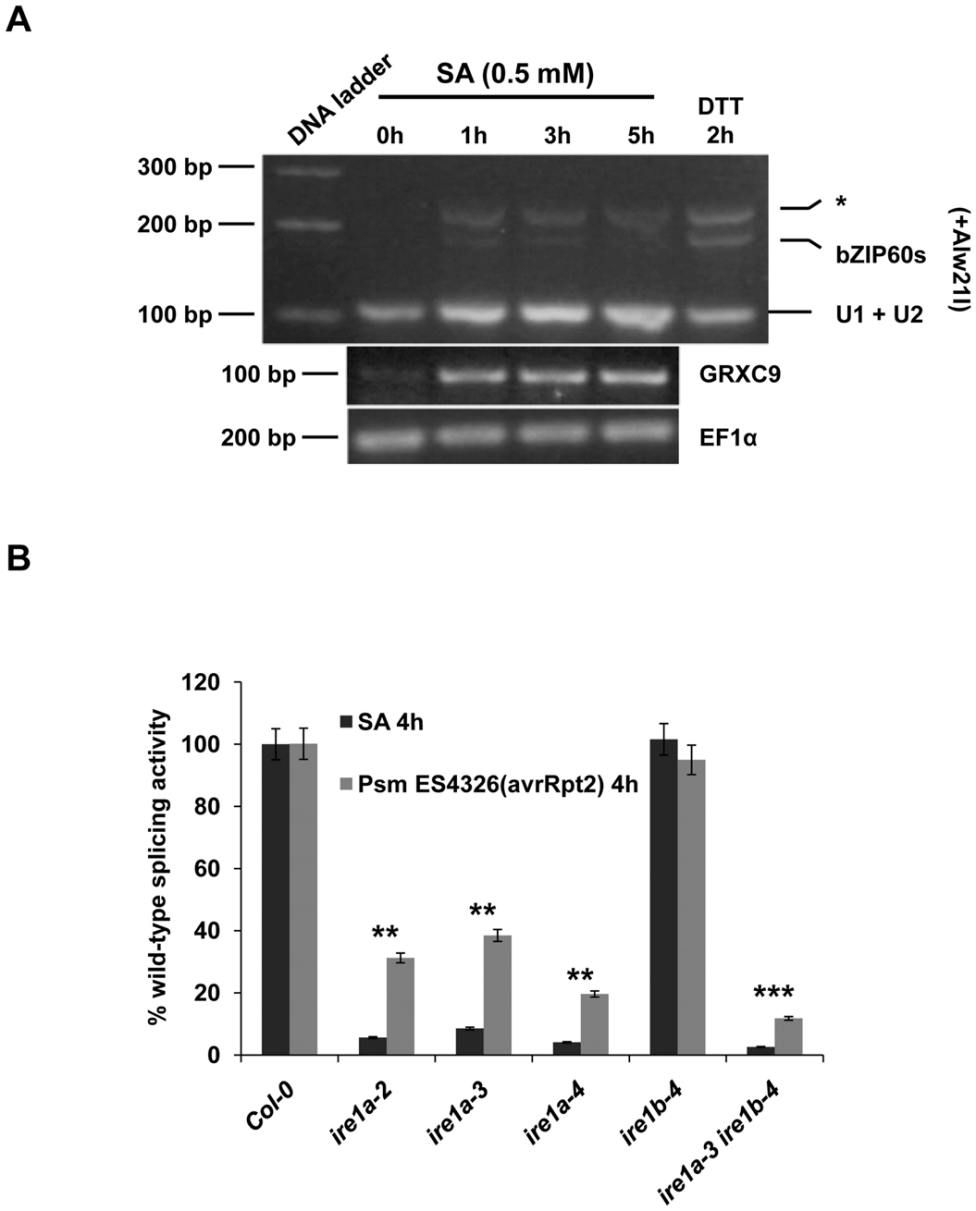
Salicylic acid stimulates bZIP60 processing. **A,** RT-PCR products derived from bZIP60 mRNA were digested with Alw21I and resolved by gel electrophoresis in agarose (3.5% p/v). RNA samples were obtained from seedlings (6-days-old) of wild-type plants treated with salicylic acid (SA) for the indicated time. As a positive control we used a RNA sample obtained from seedlings treated with DTT (5 mM) for 2 hours. GRXC9 gene expression served as control for the action of SA at transcriptional level [29], [57]. Elongation factor 1 alpha (EF1α) gene expression served as a control. **B,** Pathogen infection and SA induce bZIP60 splicing in IRE1a-dependent manner. cDNA was made from the leaf tissue of 3-week-old plants of the indicated genotypes infected with *Psm* ES4326(avrRpt2) or sprayed with SA for 4 hours. Ratios of fold induction of spliced and unspliced bZIP60 in the listed genotypes are plotted, while ratio of Col-0 was set as 100%. Statistical analysis was performed using Student's *t*-test, **, *p*<0.01, ***, *p*≤0.001. All the experiments were performed at least three times with similar results.

Given that SA can induce UPR and IRE1 is required for efficient PR1 secretion as well as mounting effective SAR, we further investigated whether SA is a signal capable of activating the IRE1/bZIP60 signaling pathway. We examined bZIP60 mRNA processing (Figure 6A) in wild-type plants treated with 0.5 mM SA over the course of 5 hours. Such treatment also stimulates the transcription of *GRXC9*, a gene known to be early induced by SA [29]. We demonstrated that bZIP60 processing can be detected as early as one hour post SA treatment and the bZIP60s form persisted up to 5 hours. However, bZIP60s was completely absent in SA-treated *ire1a-2 ire1b-4* plants (Figure S11). Next, we examined the quantitative requirement of IRE1a and IRE1b for bZIP60 splicing 4 hours after SA treatment and *Psm* ES4326(avrRpt2) (OD = 0.002) infection, using the qRT-PCR (see Materials and Methods). We demonstrated that pathogen- and SA-dependent bZIP60 processing is impaired in all *ire1a* mutants up to 80% and 95%, respectively (Figure 6B). In contrast, *ire1b-4* displays only a minimal reduction in bZIP60 processing (up to 5%). bZIP60 splicing was further diminished in *ire1a-3 ire1b-4* and IRE1B RNAi plants, compared to their corresponding single mutants (Figures 6B, S12). Similarly to the Tm treatment, both SA and *Psm* ES4326(avrRpt2) can readily induce bZIP60 transcript accumulation in wild-type plants, and this induction is partly affected in the *ire1a-3 ire1b-4* double mutant (Figure S13). Finally, we tested whether methyl jasmonate (MeJA), an active form of jasmonic acid, can also stimulate bZIP60 splicing. Jasmonate (JA) is considered to be another major hormone involved in plant immune responses. However, JA signaling pathway is mutually antagonistic to SA and required for resistance to necrotrophic pathogens [30]. Our results showed that MeJA failed to activate bZIP60 mRNA processing (Figure S10).

### bZIP60 is involved in plant immunity

**Figure 7:**
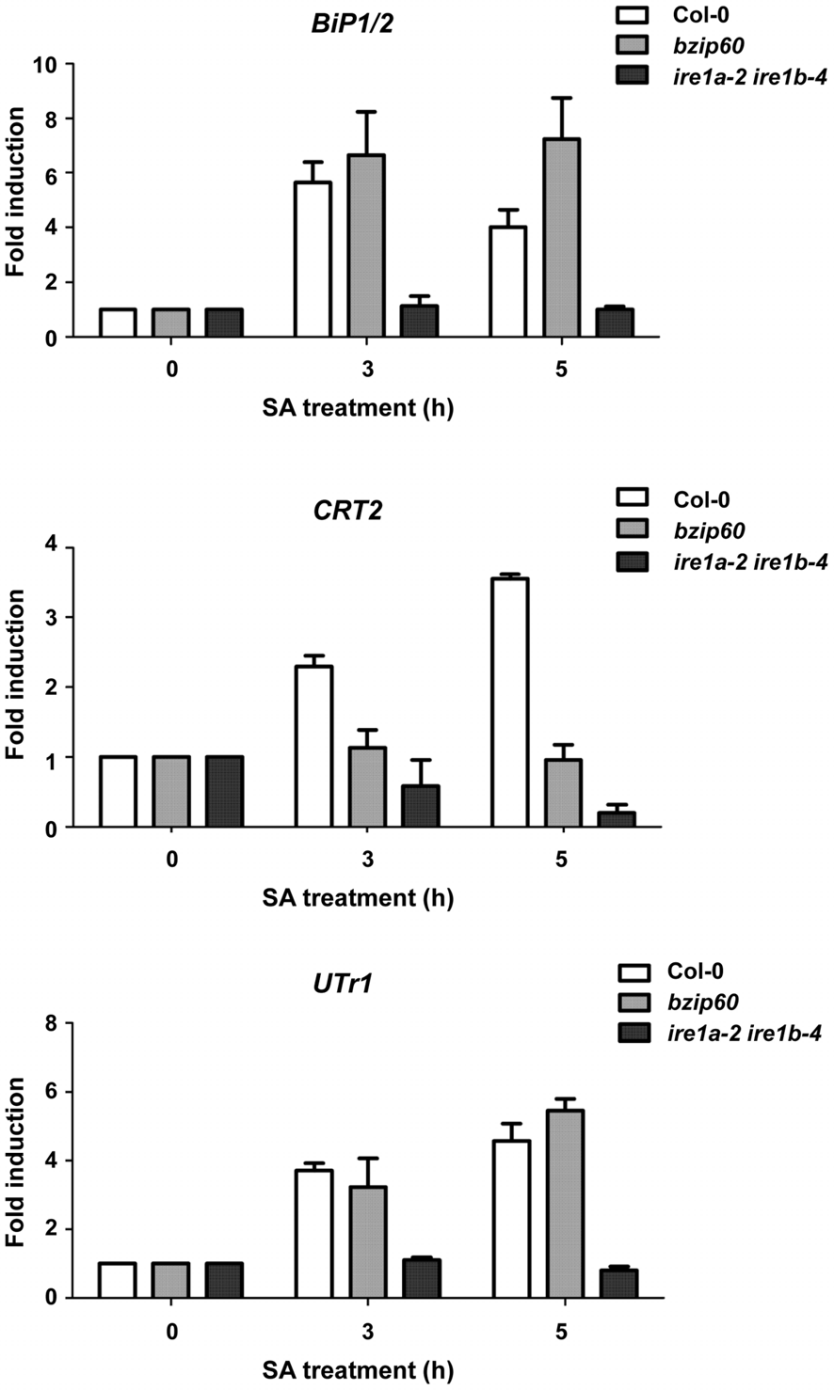
SA-induced up-regulation of UPR responding genes is altered in *ire1* and *bzip60* mutants. Plants (Col-0, *bzip60* and *ire1a-2 ire1b-4*) were treated with SA for 3 and 5 hours. RNA was extracted and quantitative PCR was performed for BiP1/2, calreticulin 2 (CRT2) and the UDP-glucose transporter (UTr1). The results were normalized against a housekeeping gene (putative clathrin adaptor). Experiment was performed at least three times with similar results.

Previously, we illustrated that SA can promote the up-regulation of UPR responsive genes [8], [9]. Does this induction require the IRE1/bZIP60 branch of the UPR signaling pathway? We demonstrated that SA-dependent induction of BiP1/2, CRT2 and UTr1 was abolished in the plants lacking both members of functional IRE1 (Figure 7). However, *bzip60* plants showed a clear effect only on the CRT2 transcript. In addition, both total and secreted PR1 were unaffected in the *bzip60* mutant plants (Figure S14). These results suggest that IRE1 proteins play a role in response to SA in a manner that involves not only bZIP60 but also additional unknown clients, perhaps other transcription factors.

**Figure 8:**
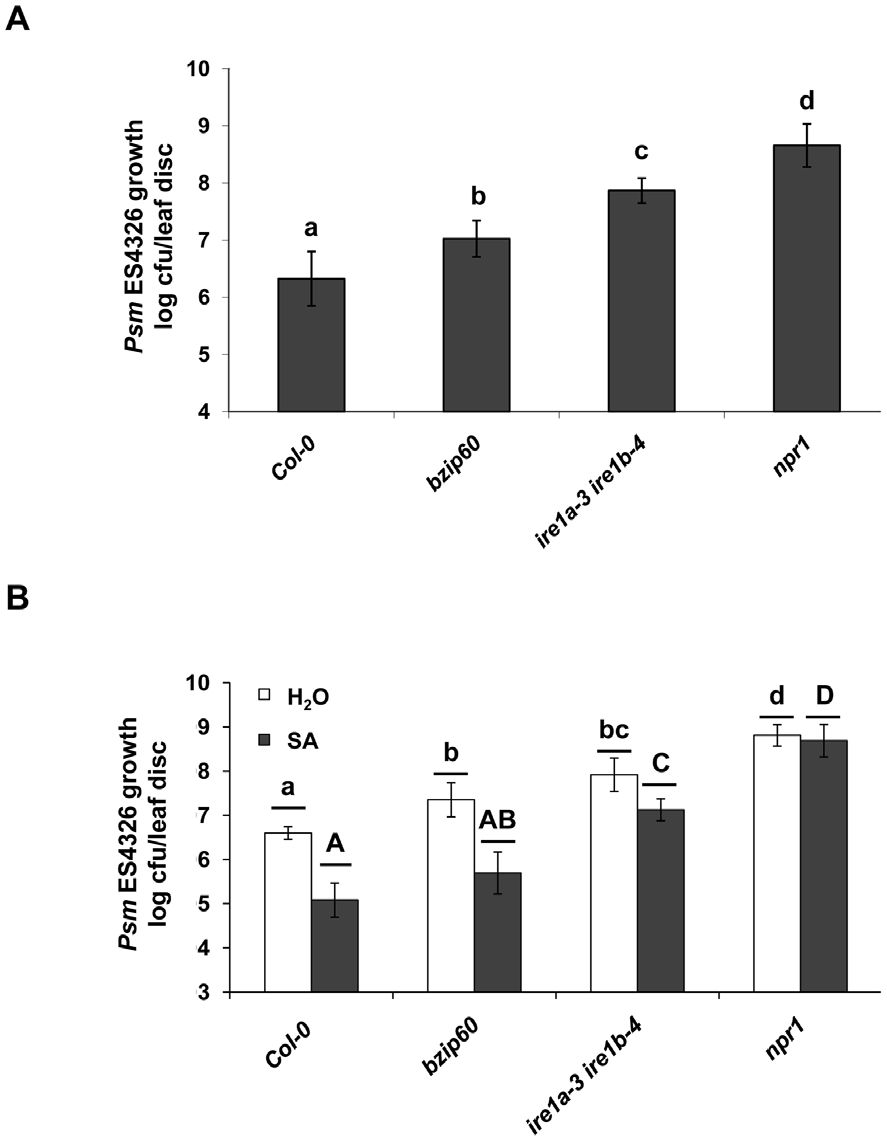
bZIP60 is involved in plant defense. **A,** Col-0, *bzip60*, *ire1a-3 ire1b-4* and hypersusceptible *npr1* mutant were infected with *Psm* ES4326 (OD = 0.0002). Bacterial growth (colony forming units – cfu/leaf disc, expressed on a log scale) was quantified in the leaves of indicated genotypes at 3 dpi. Error bars represent 95% confidence intervals of the mean (*n* = 8). Bars connected by the same letter did not differ from each other at *p*<0.05 (Tukey's HSD tests). **B,** Chemical SAR was established by treating Col-0, *bzip60*, *ire1a-3 ire1b-4* and hypersusceptible *npr1* mutant with 1 mM SA or mock (water) 16 hours prior to *Psm* ES4326 (OD = 0.001) infection. Bacterial growth was monitored 3 days post inoculation. Error bars represent 95% confidence intervals of the mean (*n* = 8). Bars within a class connected by the same letter (lowercase for water treatment; uppercase for SA treatment) did not differ from each other at *p*<0.05 (Tukey's HSD tests). All the experiments were performed at least three times with similar results.

The partial involvement of bZIP60 in SA-induced UPR genes leads to the question about its role in plant immunity. To shed light on this matter, we infected *bzip60* mutant with *Psm* ES4326 and monitored bacterial growth over the course of three days. The *bzip60* mutant plants exhibited an enhanced susceptibility compared to wild-type plants (Figure 8A), even though it was lower than the effect observed on the IRE1 mutants. Finally, we tested whether *bzip60* can mount effective SAR by infiltrating *Psm* ES4326 in plants 16 hours after SA treatment. We observed a five-fold higher bacterial growth in the *bzip60* mutant compared to wild-type plants (Figure 8B). Taken together, these data show that bZIP60 plays a role in plant immunity but is not a sole IRE1 client involved in defense responses.

## Discussion

Understanding the specific roles of UPR in plant immune responses is a great challenge as plant cells are pluripotent and have sophisticated mechanisms to prioritize and balance the different physiological processes when facing external challenges. In the current study we genetically dissected the additive as well as specific functions of both *IRE1* genes upon biotic and Tm-induced ER stresses. We showed that the SA-mediated induction of downstream ER-responsive genes and UPR marker genes as well as the secretion of antimicrobial PR proteins are more severely affected in *ire1a* mutants as compared to *ire1b* (Figure 2). Furthermore, while both *IRE1* genes are required in establishing effective SAR, IRE1a appears to play a predominant role in this process under the conditions tested (Figure 3). However, in response to Tm-induced ER stress, we demonstrated additive functions of IRE1a and IRE1b (Figures 1, 5) with IRE1b being the more substantial contributor. The differential functions of IRE1a and IRE1b may be a consequence of their dissimilar protein kinase activation loops [24]. In contrast to the plant proteins, the mammalian IRE1α and IRE1β, while having very similar protein kinase activation loops, appear to have endonuclease domains cleaving distinct RNA targets [31]. IRE1α can autoregulate its own mRNA abundance through an endonucleolytic event [32], while IRE1β attenuates its own translation through inducing degradation of 28S ribosomal RNA by an endonucleolytic event [33]. In mice, deletion of *IRE1*α is embryo lethal, while deletion of *IRE1*β is viable, but results in increased sensitivity to colitis induced by dextran sodium sulfate [34]. Together with our results from this study, it is reasonable to postulate that mechanisms of mammalian and Arabidopsis UPR are more complex than those in yeast [25], [35], since these organisms evolved an additional *IRE1* gene as well as other UPR sensors to perform diverse functions. Moreover, it has been shown that the Arabidopsis *IRE1a* and *IRE1b* genes have largely overlapping expression patterns [23], but IRE1b transcript appears to be more abundant in the floral tissue [36]. Similarly, the mammalian IRE1α is ubiquitously expressed [37], whereas expression of IRE1β is limited to the epithelium of the gastrointestinal tract [34].

The Arabidopsis bZIP60 was found due to the conserved hairpins in its mRNA, which are known to be critical for the IRE1-mediated unconventional splicing of HAC1 and XBP-1 mRNA (Figures S7, S8) [25], [26]. Interestingly, while the human IRE1 enzymes are able to splice the yeast HAC1 mRNA *in vitro* [37], it is not spliced in Arabidopsis protoplasts upon Tm treatment [23].

bZIP60 functions in abiotic and biotic stresses have been previously demonstrated. Over-expression of bZIP60 yields tolerance to salt stress in Arabidopsis [38]. In addition, an up-regulation in the expression of bZIP60 and BiP2 is observed when plants are exposed to salt-induced UPR [39], [40]. However, bZIP60 mRNA processing is not induced upon salt stress (Figure S10) [25]. Recent reports have also suggested that there is a link between bZIP60 and pathogen attacks as Arabidopsis and *N. benthamiana* plants infected with viruses showed an induction of bZIP60 [41], [42]. Silencing of *NbbZIP60*, an ortholog of *bZIP60* in *Nicotiana benthamiana* that plays a role in ER stress, resulted in enhanced susceptibility to a non-host pathogen [43]. Finally, analyses of public transcriptomic data in the Genevestigator database (see https://www.genevestigator.com, [44]) show a significant accumulation of bZIP60 mRNA in plants infected with different pathogens.

Previously, Iwata et al. 2009 speculated that AtbZIP60 might be activated by a proteolytic cleavage. However, recently published work from the same laboratory [26], another report [25] and our study all confirmed that the active form of the bZIP60 protein is synthesized from the mRNA spliced by IRE1 endonucleases. We demonstrate that the induction and splicing of bZIP60 can also be activated in response to the immune signal SA and to a bacterial pathogen challenge. Previously, it has been shown that both IRE1a and IRE1b can splice bZIP60 mRNA *in vitro* [25]. We employed a range of biotic (pathogen infection and SA) and abiotic (DTT, Tunicamycin, heat and CPA) stresses to understand the differential roles of IRE1a and IRE1b in bZIP60 splicing. Our quantitative splicing data lend some evidence for a potential preferential requirement of IRE1a in the immune-induced bZIP60 processing. Conversely, IRE1b participates almost exclusively in bZIP60 splicing during UPR induced by Tm- or DTT-induced ER stresses. IRE1-mediated bZIP60 splicing is different than the action of other bZIP family members involved in UPR sensing and signaling, such as bZIP17 and bZIP28. Both of these bZIP factors possess two protease cleavage sites (S1P and S2P) and undergo UPR stress-triggered proteolytic cleavage to produce an active protein that is in turn translocated to the nucleus [35], [45], [46].

It is not completely clear how bZIP60 is mechanistically involved in plant immunity and what other IRE1 clients may function in concert with bZIP60. Since SA-induced bZIP60 mRNA processing occurs prior to secretion of PR1, it is reasonable to hypothesize that bZIP60 is, at least partially, involved in the upregulation of the secretory machinery during plant immune response to accommodate to the massive production of antimicrobial proteins [9]. Similarly, the mammalian XBP-1 has been found to be required for the development of plasma cells from which large amounts of immunoglubolin proteins are secreted [14], [15]. Recently, IRE1-spliced XBP-1 transcript in nematode *C. elegans* was detected within 4 hours of exposure to *P. aeruginosa* [16]. Infection of the *xbp-1* mutant with *P. aeruginosa* leads to disruption of ER morphology and larval lethality. Interestingly, this lethal phenotype is not due to excessive proliferation of *P. aeruginosa* but rather activation of a receptor PMK-1 [16]. Thus, it was proposed that XBP-1 suppresses the detrimental effect of PMK-1 activation during the immune response but does not facilitate the elimination of the pathogen.

The IRE1-XBP-1/Hac1/bZIP60 is the most conserved branch of the UPR and has been suggested to play crucial roles in a wide range of biological processes including development, metabolism, inflammation and immunity [47], [48]. Our results show that IRE1/bZIP60 play distinct roles in both abiotic and biotic stresses. Our quantitative recovery assay showed a significant decrease in the survival of *bzip60* seedlings on Tm as compared to wild-type. However, this rescue rate was still higher than that of *ire1a-3 ire1b-4* double knock-out plants (Figure S15). These data are in agreement with the recent expression profiling study that demonstrated a large, but not complete, overlap in genes differentially regulated by bZIP60 and IRE1a/IRE1b [26]. Similarly, the immune defect in neither *ire1a ire1b* double mutant nor *bzip60* plants is as profound as that observed in the *npr1* mutant. It is possible that other branches of the UPR may also participate in plant immunity. In this regard, some of the differences observed between the phenotypes of *ire1a ire1b* and *bzip60* suggest the existence of other IRE1 functions, which are independent of bZIP60 signaling, under both abiotic and biotic stresses.

Interestingly, similar observations have been previously made in other systems. Although the mammalian IRE1α acts mainly via XBP-1 splicing, in pancreatic B-cells, glucose can enhance IRE1α phosphorylation and augment insulin biosynthesis without increase in XBP-1 splicing [49]. In Drosophila, IRE1 can degrade specific mRNAs undergoing translation at the ER membrane and halt protein synthesis [50]. Recently, Feng et al. demonstrated that in the absence of ER stress, *Aspergillus fumigatus* Ire1 controls dual signaling circuits that are both Hac1-dependent and Hac1-independent [51]. Our study in plants highlights a complex regulatory mechanism of UPR which may have been evolved to suit the sessile nature of plants in response to a variety of stimuli.

## Methods

### Mutants and transgenic lines used in this study

All mutants reported below were obtained from the Arabidopsis Biological Resource Center and are in Col-0 background, with the exception of *ire1b-4* that is in Col-3 background. We isolated a homozygous *bzip60* (Col-0; SALK_050203) mutant line. For *IRE1a*, we acquired three independent homozygous T-DNA insertion lines: *ire1a-2* (SALK_018112), *ire1a-3* (WiscDsLox420D09) and *ire1a-4* (SAIL_1256_F04) and showed that all three alleles were characterized by a complete loss of IRE1a transcript. *ire1a-1* line (SALK_010332) has been previously reported by Lu and Christopher [52] and shown to contain residual levels of IRE1a transcript; thus, we chose not to use it in this study. For *IRE1b*, we also obtained three independent T-DNA insertion lines (*ire1b-2*, SAIL_252_A05; *ire1b-3*, SALK_018150 and *ire1b-4*, SAIL_238_F07). Nagashima et al. (2011) recently reported *ire1b-1* (GABI_638B07), thus we maintained a continuous nomenclature of the additional alleles in our report. We were unable to procure *ire1b-2* and *ire1b-3* homozygous mutants. After self-fertilizing plants heterozygous for a T-DNA insertion, populations of 1/3 wild-type plants and 2/3 heterozygous plants were recovered in multiple attempts. We tested pollen viability by Alexander staining method, as well as seed set and seed germination rates but found no defect in heterozygous *IRE1b/ire1b* plants compared to the wild-type. We reached the conclusion that homozygous *ire1b-2* and *ire1b-3* plants are unviable but the reason for this is unclear. A similar observation was described in two other reports [26], [52]. Nagashima et al. also reported failure to complement *ire1b-2* and *ire1b-3* by a genomic *IRE1b* sequence, which indicates that the truncated IRE1b-2 and IRE1b-3 proteins might be toxic to the cell and result in lethality.

We were able to obtain *ire1b-4* homozygous mutant plants and we did not detect the presence of a full-length transcript in these plants.

To acquire double *ire1* mutants, we crossed *ire1a-2* and *ire1a-3* to *ire1b-4* and obtained two independent double mutant lines: *ire1a-2 ire1b-4* and *ire1a-3 ire1b-4*.

In order to obtain additional genetic tools to study IRE1b function, we also created stable RNAi transgenic plants. We identified a part of the IRE1b sequence, located within the 3′ region of the transcript that shared no homology with any other Arabidopsis gene. We amplified a 370 bp-long fragment using Gateway-adapted PCR primers Ire1b-RNAi-F and Ire1b-RNAi-R2 and cloned it into pDONR207. The resulting pENTR207-IRE1b-RNAi clone was next confirmed by sequencing and recombined into the plant expression vector pJawohl8 RNAi (kind gift of I. E. Somssich, MPI for Plant Breeding Research, Cologne, Germany). Col-0 and *ire1a-2* plants were transformed with the obtained construct pJawohl8 IRE1b-RNAi using Agrobacterium-mediated floral dip method [53]. Resulting T_1_ and T_2_ seedlings were selected on BASTA. In the T_3_ generation, 25–30 independent lines per genetic background were assessed for their zygocity as well as basal and induced IRE1b transcript levels in leaves (Figure S2). Two homozygous lines with the most profound reduction in IRE1b transcript levels were selected for further analyses.

### Plant growth conditions

For the RNA and protein sampling and pathogen infection, seeds were incubated for 72 h at 4°C and grown on MetroMix 360 soil under long day conditions (16 h light/8 h dark) at 65% humidity for three weeks.

For Tm recovery, seeds were briefly washed in 70% Ethanol and placed in 2% Plant Preservative Mixture (PPM) for 72 h at 4°C. Subsequently, PPM was discarded and seeds were placed in sterile 0.1% Difco agar solution, and spread thinly on solid Murashige Skoog (MS) medium supplemented with Tm (0.3 µg/mL; Sigma) for 72 h at 22°C. After Tm exposure, 25 seeds per genotype were transferred to 0.8% agar MS medium supplemented with Ampicillin (50 µg/mL) and grown on horizontal plates. After 10 days, recovery was recorded. Original Tm plates were kept and checked to ensure that there was no recovery of the remaining seedlings. For all other chemical treatments, 6-days-old seedlings grown in liquid 0.5× MS were used and chemicals added to media at indicated concentration for indicated times.

### RT-PCR and bZIP60 splicing assay

Arabidopsis seedlings or detached leaves were harvested in liquid nitrogen. RNA was extracted from each sample using TRIzol reagent (Invitrogen) and treated with DNase I. cDNA was synthesized using a SuperScript II first-strand RT-PCR kit (Invitrogen). The primers used in this study are listed in Table S1.

For gel-based bZIP60 splicing assay, PCR conditions for amplification were: initial denaturation: 5 min at 95°C; 45 s at 95°C, 15 s at 55°C and 30 s at 72°C during 35 cycles; final extension 5 min at 72°C. Subsequently, PCR products were digested using Fast Digest® Alw21I enzyme restriction (Thermo Scientific Fermentas) following manufacturer instructions. Digested products were resolved by gel electrophoresis on agarose-1000 (3.5% p/v) (Invitrogen) using TAE 1X as running buffer.

For q-PCR based assay, transcript abundance was quantified using bZIP60u or bZIP60s specific primers (Table S1) using the SYBR GREEN PCR Master Mix (Applied Biosystems) in a RealPlex S MasterCycler (Eppendorf). Wild-type, *ire1a-2*, *ire1a-3*, *ire1a-4*, *ire1b-4* and *ire1a-3 ire1b-4* plants were treated with Tm for 0, 2 and 5 hours. Since bZIP60 is readily activated by Tm treatment [54], we next calculated fold induction of the bZIP60u or bZIP60s transcripts over their basal levels. We subsequently plotted ratios between the fold induction of the spliced vs. unspliced bZIP60 forms by adjusting the wild-type ratio as 100% (Figures 5B, S9). bZIP60 transcript analysis presented in Figure S13 was performed with a different set of primers, bZIP60_FWD and bZIP60_REV (Table S1) that amplify both unspliced and spliced bZIP60 forms.

### Stress Assays and Hormones Treatment

15-day-old Arabidopsis seedlings grown in solid MS medium were treated with liquid MS medium alone and incubated at 37°C (heat stress) or 4°C (cold stress) for the indicated time. To test salt and osmotic stress, seedlings were treated with liquid MS medium containing 150 mM NaCl (salt stress) or 300 mM Mannitol (osmotic stress) for indicated time. To evaluate the role of hormones and chemicals involved in biotic and abiotic stresses, 6-day-old seedlings grown in liquid MS media were treated with SA (0.5 mM), MeJA (30 µM), Tm (5 µg/mL), DTT (5 mM), CPA (100 µg/mL) or Thapsigargin (500 nM). Seedlings were treated for the indicated times.

### Bacterial Strains, Plant Inoculation Procedures, and Bacteria Growth Measurements

Infection of Arabidopsis plants with *Pseudomonas syringae* pv. *maculicola* (*Psm*) ES4326 was performed as described previously [55]. To test for enhanced disease susceptibility, a bacterial suspension of OD_600_ = 0.0002 was infiltrated into 2–3 leaves per plant and 12 plants/genotype. Bacterial growth was quantified 3 days later. To test for SAR, plants were pre-treated with 1 mM SA or mock (H_2_O) spray 16 hours prior to infection and subsequently inoculated with *Psm* ES4326 (OD_600_ = 0.001) into 2–3 leaves per plant and 12 plants/genotype/treatment. Sampling was performed 3 days post inoculation.

### PR1 Protein Secretion

Three-week-old plants were treated with 1 mM SA for 16 hours before infiltration under vacuum in a 20 mM phosphate buffer (KH_2_PO_4_ and K_2_HPO_4_, pH = 7.4). Intercellular wash fluid was collected from equal amounts of tissue by centrifuging the infiltrated leaf samples, which were packed in a syringe, for 3 min at 1500 g. As a control, total protein was also extracted from 50 mg of leaf tissue (from 3independent plants) using a buffer described previously [9]. Secreted and total protein were run on 14% SDS-PAGE gels, transferred to a nitrocellulose membrane, and probed with a polyclonal rabbit antibody raised against a synthetic peptide matching the carboxy terminus of the Arabidopsis PR1 protein (1∶5000 dilution, 4°C, O/N) followed by goat anti-rabbit secondary antibody (Santa Cruz Biotechnology) (1∶20000 dilution, 1 hour). To confirm equal loading of total protein, Ponceau S was used to stain the total protein blot.

### Statistical analyses

Significant differences between genotypes were tested using one-tailed Student's *t*-test or ANOVA followed by the post hoc test Tukey's Honestly Significant Difference (HSD). Calculations were made using the SAS 9.2 software package (SAS Institute, Cary, NC).

## Supporting Information

Figure S1 **Schematic representation of the T-DNA insertion sites in the** ***ire1a*** **and** ***ire1b*** **mutants.** The upstream regions and genomic organizations of *IRE1a* and *IRE1b* are illustrated. Black boxes correspond to 5′ and 3′ UTRs. White boxes represent exons, while lines stand for introns. The bent arrow illustrates the predicted translation initiation sites. Asterisks symbolize stop codons. The positions of the T-DNA insertions within *IRE1a* and *IRE1b* are shown.
(TIF)

Figure S2 **IRE1b transcript accumulation in IRE1b RNAi lines in Col-0 and** ***ire1a-2*** **.** cDNA was prepared from the leaf tissues of the indicated genotypes upon treatment with SA for 4 hours and Tm for 2 hours and 5 hours as well as from untreated leaf tissues. IRE1b transcript was measured using real-time RT-PCR. Transcript abundance was normalized using UBQ5. The experiment was performed at least three times with similar results.
(TIF)

Figure S3 **Tunicamycin sensitivity of IRE1b RNAi lines.** Seedlings were grown on MS medium containing 0.3 µg/mL Tm to induce UPR for 3 days. Subsequently, seedlings were allowed to recover for additional 10 days. Percentage of recovery was plotted by calculating alive/dead seedlings of the indicated genotypes. The experiment was performed at least three times with similar results.
(TIF)

Figure S4 **PR1 secretion in IRE1b RNAi lines.** Intercellular wash fluid (IWF) was collected from 20 leaves derived from 10 plants per indicated genotype treated with SA for 16 hours. Total protein was extracted from five leaves derived from three plants per indicated genotype treated with SA for 16 hours. Accumulation of PR1 was detected by Western blots with anti-PR1 from IWF and total leaf extract. Ponceau S stain verifies equal loading. Experiments were repeated at least four times with similar results.
(TIF)

Figure S5 **Enhanced disease susceptibility test on IRE1b RNAi lines.** Bacterial growth (colony forming unit – cfu/leaf disc, expressed on a log scale) was determined from the leaves of the indicated genotypes infected with *Psm* ES4326 (OD = 0.0002). Bacterial population was assessed at 3 dpi. Hypersusceptible *npr1* mutant was used as control. Error bars: 95% confidence interval of the mean (*n* = 8). The experiment was performed at least three times with similar results.
(TIF)

Figure S6 **Establishment of systemic acquired resistance in IRE1b RNAi lines.** All the genotypes were treated with either 1 mM SA or water 16 hours prior to *Psm* ES4326 infection (OD = 0.001). Bacterial growth was monitored 3 days post inoculation. Hypersusceptible *npr1* mutant was used as control. Error bars: 95% confidence interval of the mean (*n* = 8). The experiment was performed at least three times with similar results.
(TIF)

Figure S7 **Prediction of stem-loop structures observed in XBP-1, HAC1 and bZIP60 mRNA.** The conserved nucleotides essential for splicing of XBP-1, HAC1 and bZIP60 mRNAs are boxed in red.
(TIF)

Figure S8 **Sequence prediction of spliced and unspliced bZIP60 forms.** **A,** Nucleotide sequence of unspliced bZIP60 mRNA forming two hairpin structures. Spliced portion of the sequence (23 bp) is marked in red (Top). Nucleotide sequence of unspliced and spliced bZIP60 cDNAs around the splicing sites (Bottom). **B,** Schematic representations of bZIP60u and bZIP60s cDNAs indicating positions of stop codons in both transcripts. **C,** Schematic representations of bZIP60u and bZIP60s protein variants. The amino acid sequence corresponding to the putative transmembrane domain (TM) in bZIP60u is highlighted in red. A putative Nuclear Localization Signal (NLS) in bZIP60s is marked.
(TIF)

Figure S9 **Quantitative measurement of bZIP60 Tm-induced splicing activity in IRE1b RNAi lines.** cDNA was made from the leaf tissue of the indicated genotypes, non-treated or injected with 0.5 µg/mL Tm for 2 hours and 5 hours. Ratios of fold induction of spliced and unspliced bZIP60 are plotted, while setting ratio of Col-0 as 100%. The experiments were performed at least three times with similar results.
(TIF)

Figure S10 **bZIP60 processing upon diverse abiotic and biotic stresses.** RT-PCR products derived from bZIP60 mRNA were digested with Alw21I and resolved by gel electrophoresis in agarose (3.5% p/v). RNA samples were obtained from seedlings (6-day-old) of wild-type plants exposed to indicated treatments. C corresponds to a RNA sample obtained from seedlings treated with DTT (5 mM) for 2 hours (positive control to visualize splicing). L stands for DNA ladder. Expression levels of HSP20, CBF3, RCI2, HHP1, and LOX2 served as controls for the action of heat, cold, salt, mannitol and MeJA, respectively. Elongation factor 1 alpha (EF-1α) gene expression served as a control.
(TIF)

Figure S11 **bZIP60 processing upon SA treatment in wild-type and** ***ire1a ire1b*** **double mutant plants.** RT-PCR products derived from bZIP60 mRNA were digested with Alw21I and resolved by gel electrophoresis in agarose (3.5% p/v). RNA samples were obtained from 6-day-old seedlings treated with SA for 3 hrs. Expression levels of IRE1A and IRE1B were determined in the same samples. No cDNA was used as a negative control for background amplification. Elongation factor 1 alpha (EF-1a) gene expression served as a loading control.
(TIF)

Figure S12 **Pathogen infection- and SA-dependent bZIP60 splicing activity.** cDNAs were made from the leaf tissues of the indicated genotypes, untreated or treated with *Psm* ES4326(avrRpt2) and SA for 4 hours. Ratios of fold induction of spliced and unspliced bZIP60 are plotted, while adjusting ratio of Col-0 as 100%. All the experiments were performed at least three times with similar results.
(TIF)

Figure S13 **bZIP60 transcript accumulation in Col-0 and various** ***ire1*** **mutants upon SA or pathogen treatment.** cDNA was prepared from the leaf tissues of the indicated genotypes upon treatment with SA or *Psm*ES4326(avrRpt2) for 4 hours as well as from untreated leaf tissues. bZIP60 transcript was measured using real-time RT-PCR. Transcript abundance was normalized using UBQ5. The experiment was performed at least three times with similar results.
(TIF)

Figure S14 **Total and secreted PR1 protein accumulation in** ***bzip60*** **plants.** Intercellular wash fluid (IWF) was collected from 20 leaves derived from 10 plants per indicated genotype treated with SA 16 hours prior to sampling. Total protein was extracted from five leaves derived from three plants per indicated genotype. Accumulation of PR1 was detected by Western blots with anti-PR1 antibody in IWF and total leaf extracts from the indicated genotypes. The *npr1* mutant (*Non-expressor of PR1*) was used as control. Ponceau S stain verifies equal loading. Experiments were repeated at least four times with similar results.
(TIF)

Figure S15 **UPR stress tolerance in** ***bzip60*** **seedlings.** Wild-type, *bzip60* and *ire1a-3 ire1b-4* seedlings were grown on MS medium containing 0.3 µg/mL Tm for three days. Percentage of recovery was plotted by calculating alive/dead seedlings ten days post Tm treatment. Experiments were repeated at least three times with similar results.
(TIF)

Table S1 **List of primers used in this study.** PCR primers used for RT-PCR, q-PCR, mutants genotyping and generation of constructs described in the manuscript are listed, alongside with the loci identifiers for the corresponding genes.
(DOC)
