# Supplementary figures and images for: IRE1/bZIP60-Mediated Unfolded Protein Response Plays Distinct Roles in Plant Immunity and Abiotic Stress Responses
Source: PLoS One. 2012 Feb 16;7(2):e31944. doi: 10.1371/journal.pone.0031944 (PMC3281089; doi:10.1371/journal.pone.0031944)

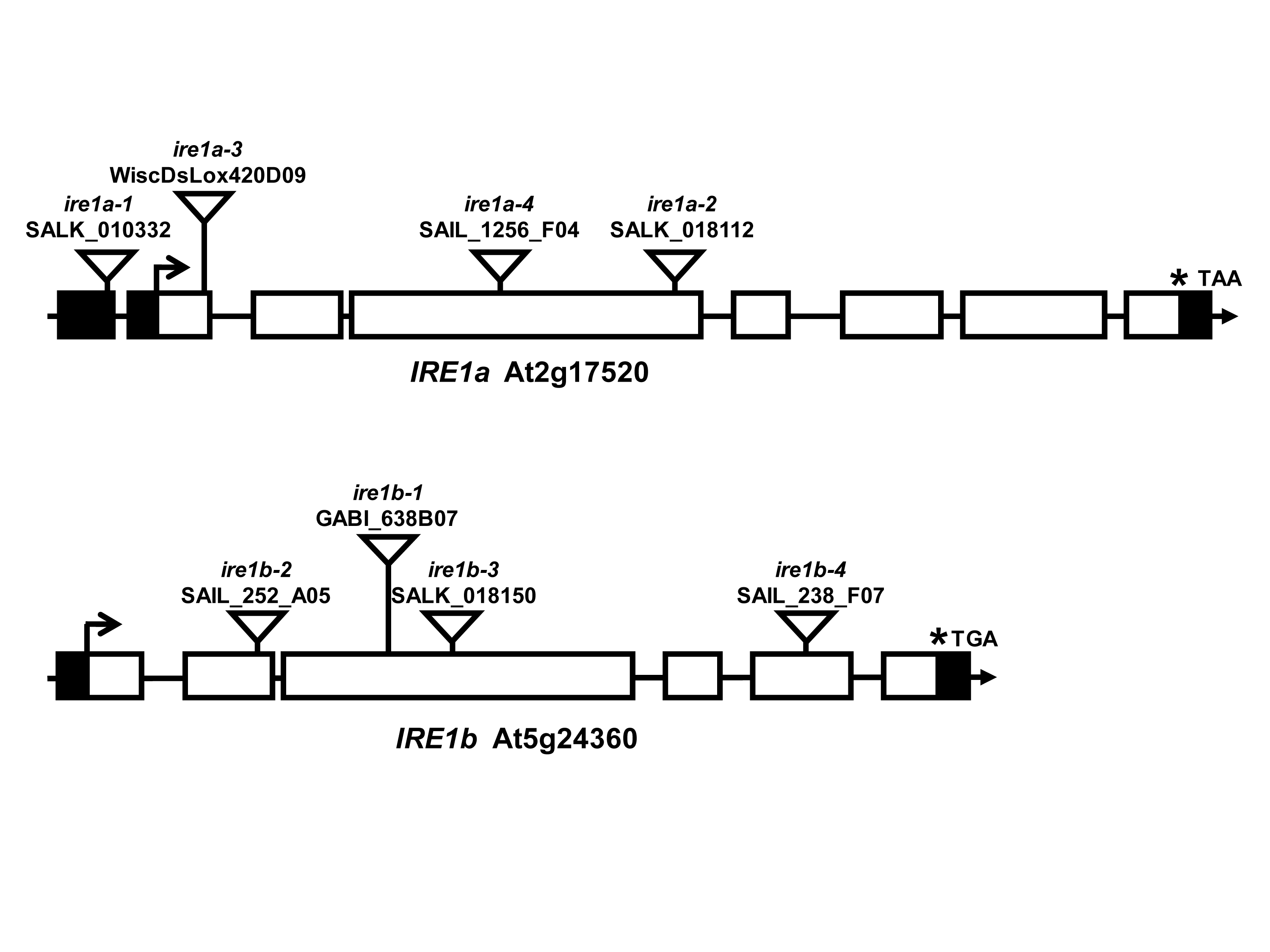

Supplement: Figure S1 — Schematic representation of the T-DNA insertion sites in the ire1a and ire1b mutants. The upstream regions and genomic organizations of IRE1a and IRE1b are illustrated. Black boxes correspond to 5′ and 3′ UTRs. White boxes represent exons, while lines stand for introns. The bent arrow illustrates the predicted translation initiation sites. Asterisks symbolize stop codons. The positions of the T-DNA insertions within IRE1a and IRE1b are shown. (TIF) [file pone.0031944.s001.tif]

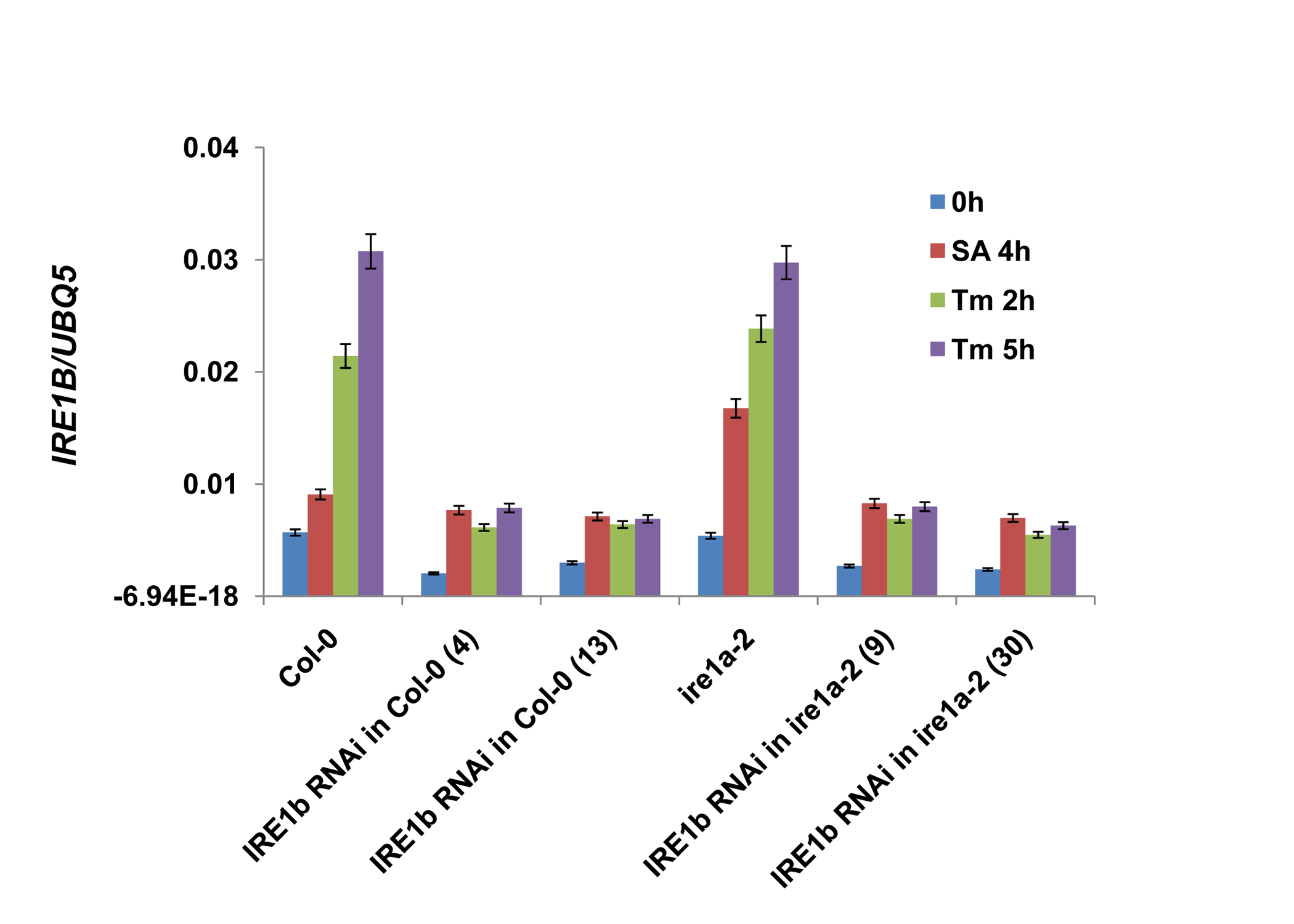

Supplement: Figure S2 — IRE1b transcript accumulation in IRE1b RNAi lines in Col-0 and ire1a-2 . cDNA was prepared from the leaf tissues of the indicated genotypes upon treatment with SA for 4 hours and Tm for 2 hours and 5 hours as well as from untreated leaf tissues. IRE1b transcript was measured using real-time RT-PCR. Transcript abundance was normalized using UBQ5. The experiment was performed at least three times with similar results. (TIF) [file pone.0031944.s002.tif]

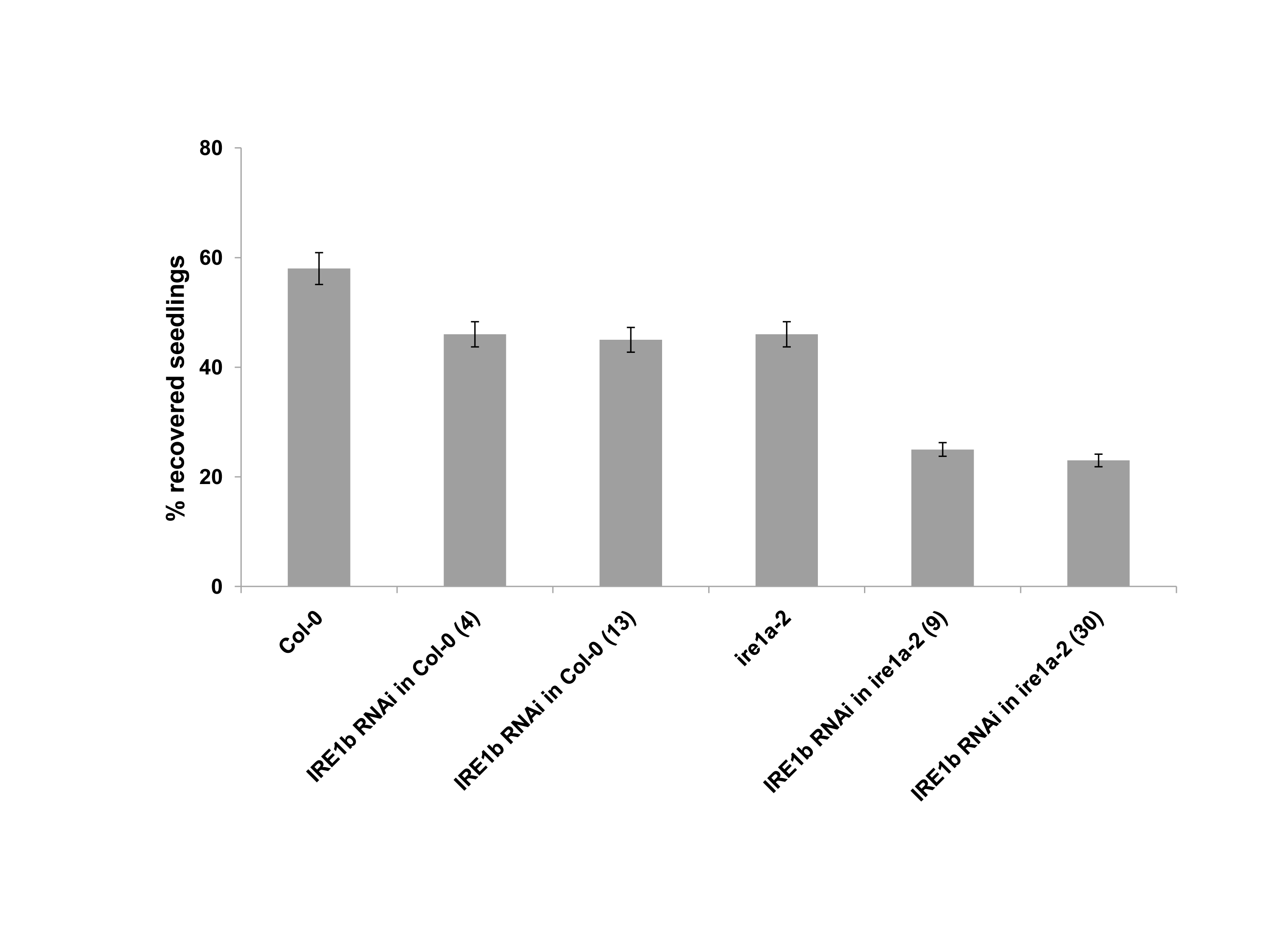

Supplement: Figure S3 — Tunicamycin sensitivity of IRE1b RNAi lines. Seedlings were grown on MS medium containing 0.3 µg/mL Tm to induce UPR for 3 days. Subsequently, seedlings were allowed to recover for additional 10 days. Percentage of recovery was plotted by calculating alive/dead seedlings of the indicated genotypes. The experiment was performed at least three times with similar results. (TIF) [file pone.0031944.s003.tif]

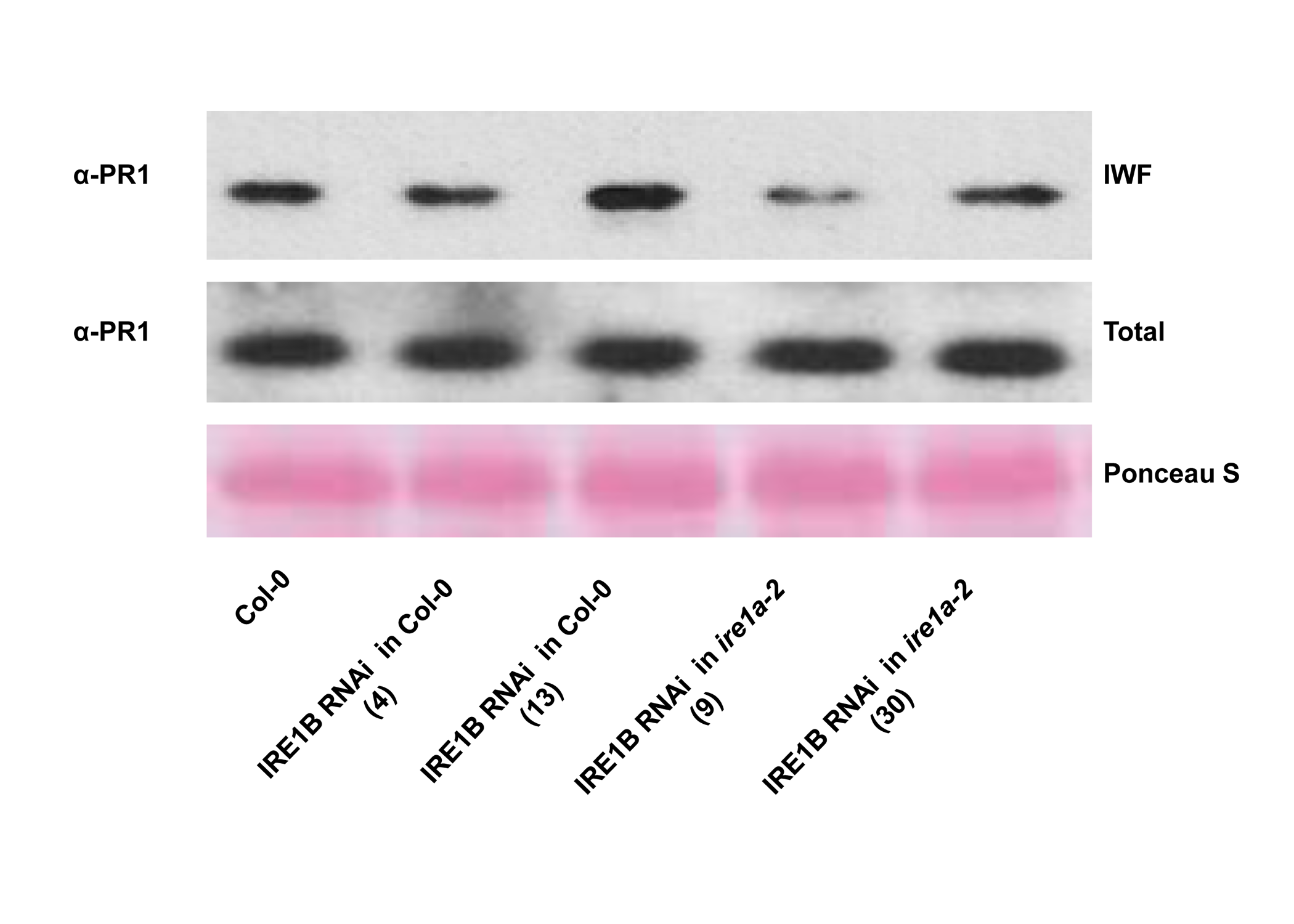

Supplement: Figure S4 — PR1 secretion in IRE1b RNAi lines. Intercellular wash fluid (IWF) was collected from 20 leaves derived from 10 plants per indicated genotype treated with SA for 16 hours. Total protein was extracted from five leaves derived from three plants per indicated genotype treated with SA for 16 hours. Accumulation of PR1 was detected by Western blots with anti-PR1 from IWF and total leaf extract. Ponceau S stain verifies equal loading. Experiments were repeated at least four times with similar results. (TIF) [file pone.0031944.s004.tif]

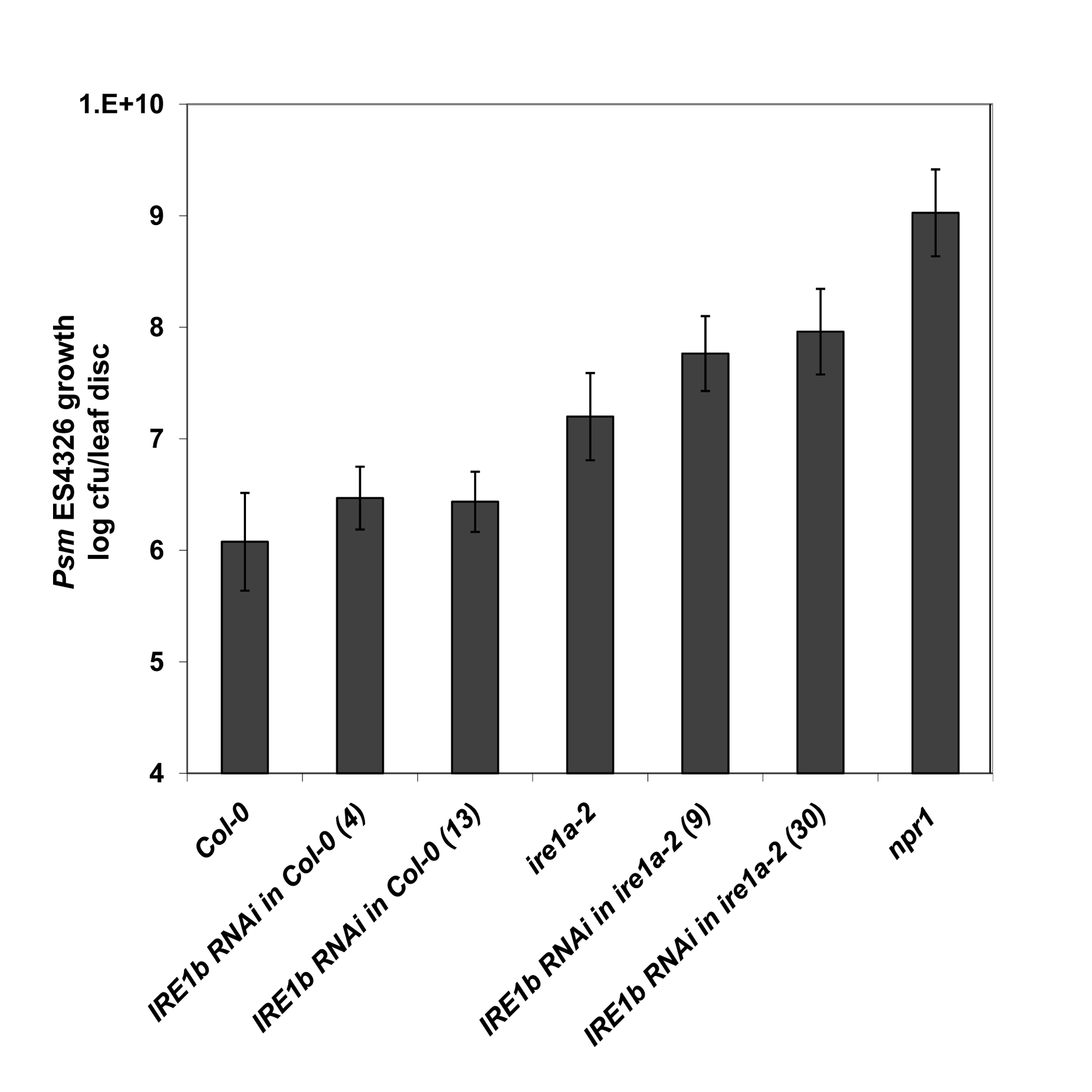

Supplement: Figure S5 — Enhanced disease susceptibility test on IRE1b RNAi lines. Bacterial growth (colony forming unit – cfu/leaf disc, expressed on a log scale) was determined from the leaves of the indicated genotypes infected with Psm ES4326 (OD = 0.0002). Bacterial population was assessed at 3 dpi. Hypersusceptible npr1 mutant was used as control. Error bars: 95% confidence interval of the mean (n = 8). The experiment was performed at least three times with similar results. (TIF) [file pone.0031944.s005.tif]

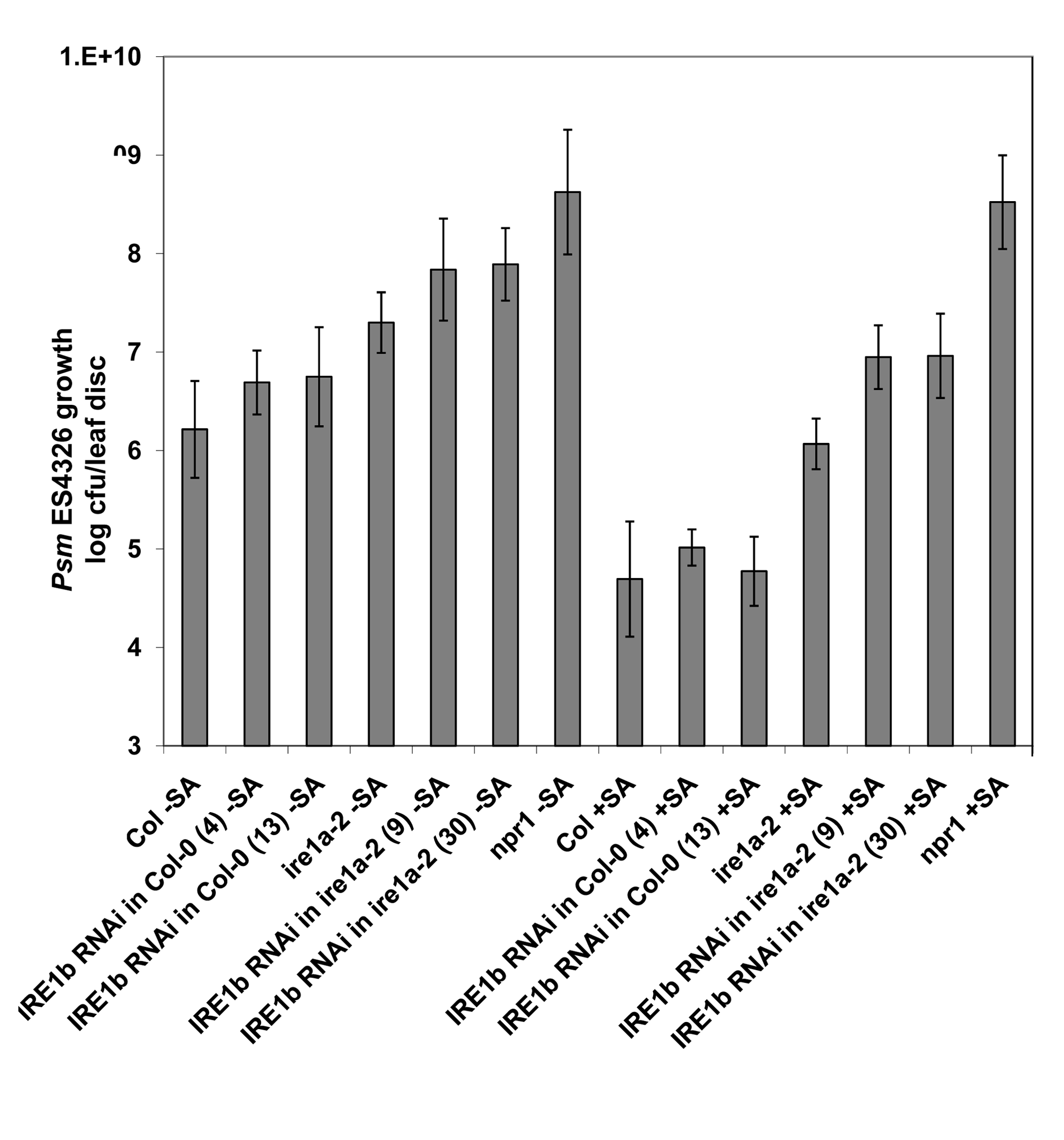

Supplement: Figure S6 — Establishment of systemic acquired resistance in IRE1b RNAi lines. All the genotypes were treated with either 1 mM SA or water 16 hours prior to Psm ES4326 infection (OD = 0.001). Bacterial growth was monitored 3 days post inoculation. Hypersusceptible npr1 mutant was used as control. Error bars: 95% confidence interval of the mean (n = 8). The experiment was performed at least three times with similar results. (TIF) [file pone.0031944.s006.tif]

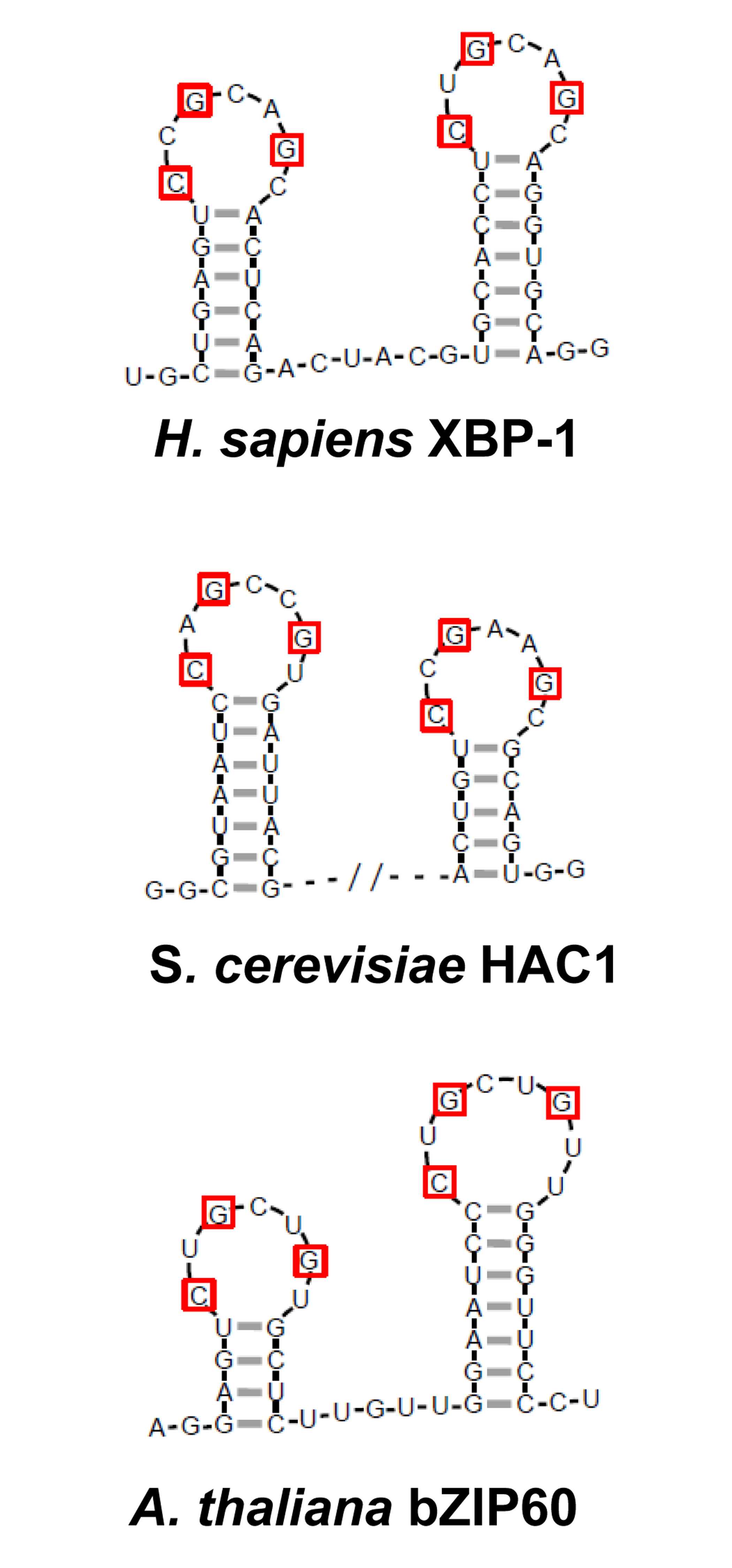

Supplement: Figure S7 — Prediction of stem-loop structures observed in XBP-1, HAC1 and bZIP60 mRNA. The conserved nucleotides essential for splicing of XBP-1, HAC1 and bZIP60 mRNAs are boxed in red. (TIF) [file pone.0031944.s007.tif]

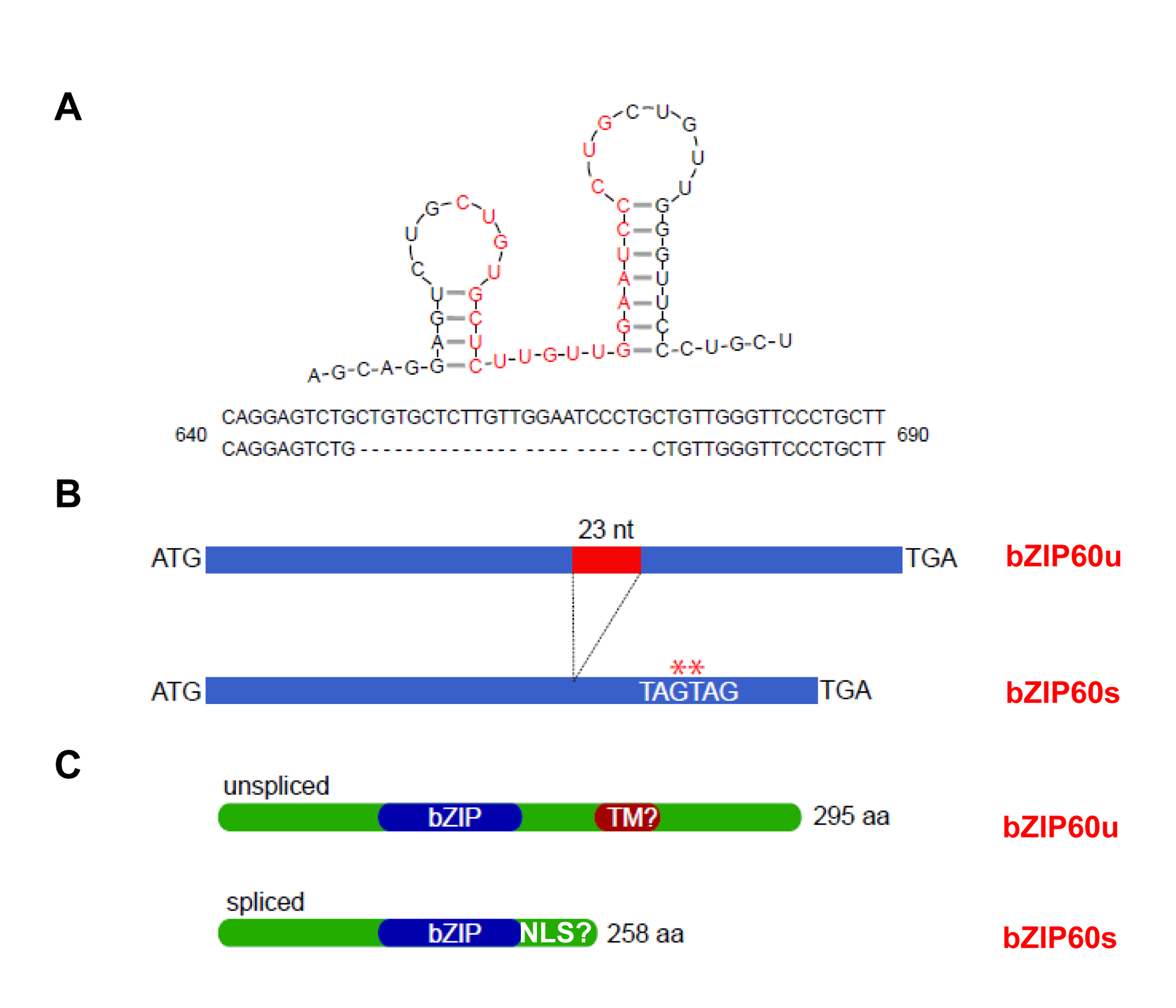

Supplement: Figure S8 — Sequence prediction of spliced and unspliced bZIP60 forms. A, Nucleotide sequence of unspliced bZIP60 mRNA forming two hairpin structures. Spliced portion of the sequence (23 bp) is marked in red (Top). Nucleotide sequence of unspliced and spliced bZIP60 cDNAs around the splicing sites (Bottom). B, Schematic representations of bZIP60u and bZIP60s cDNAs indicating positions of stop codons in both transcripts. C, Schematic representations of bZIP60u and bZIP60s protein variants. The amino acid sequence corresponding to the putative transmembrane domain (TM) in bZIP60u is highlighted in red. A putative Nuclear Localization Signal (NLS) in bZIP60s is marked. (TIF) [file pone.0031944.s008.tif]

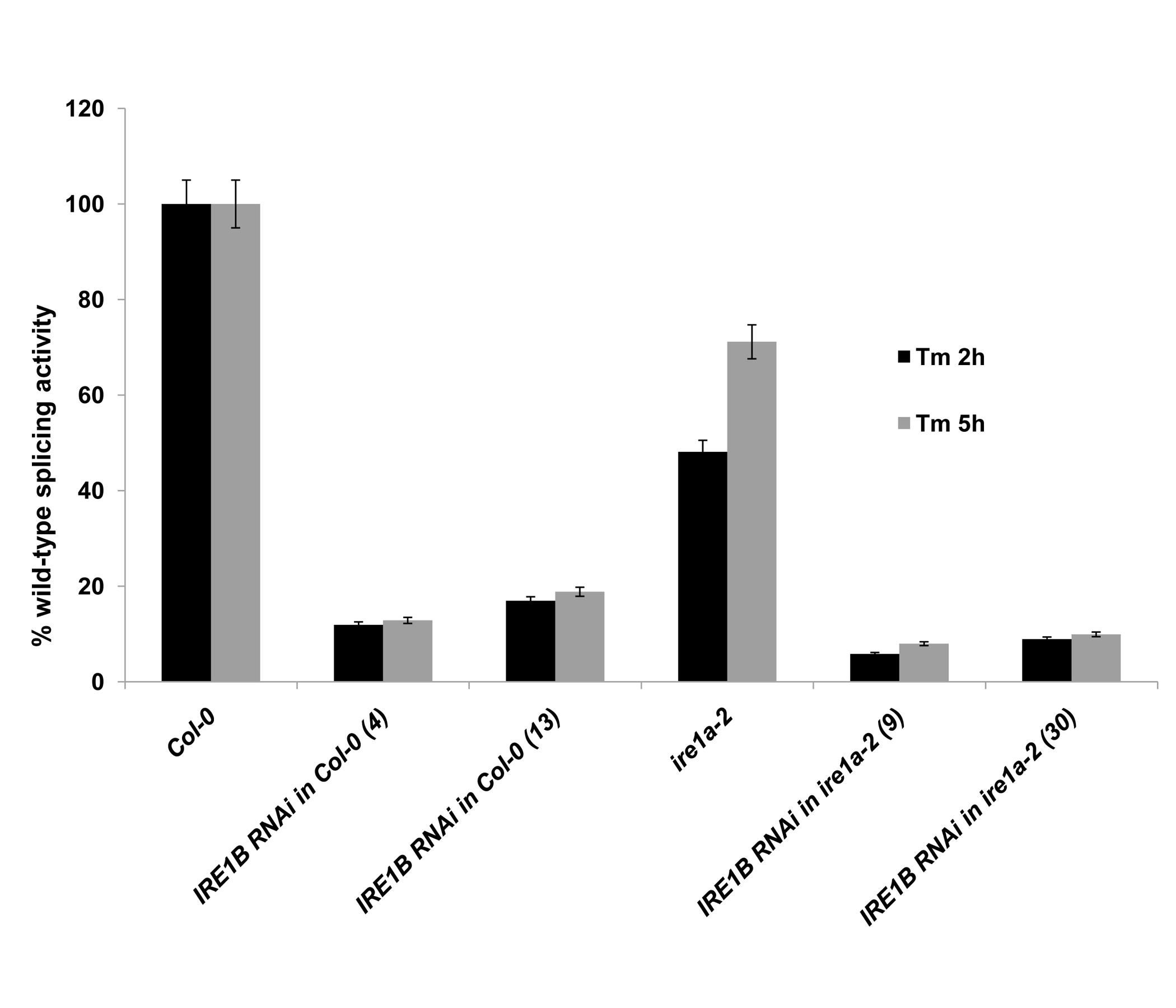

Supplement: Figure S9 — Quantitative measurement of bZIP60 Tm-induced splicing activity in IRE1b RNAi lines. cDNA was made from the leaf tissue of the indicated genotypes, non-treated or injected with 0.5 µg/mL Tm for 2 hours and 5 hours. Ratios of fold induction of spliced and unspliced bZIP60 are plotted, while setting ratio of Col-0 as 100%. The experiments were performed at least three times with similar results. (TIF) [file pone.0031944.s009.tif]

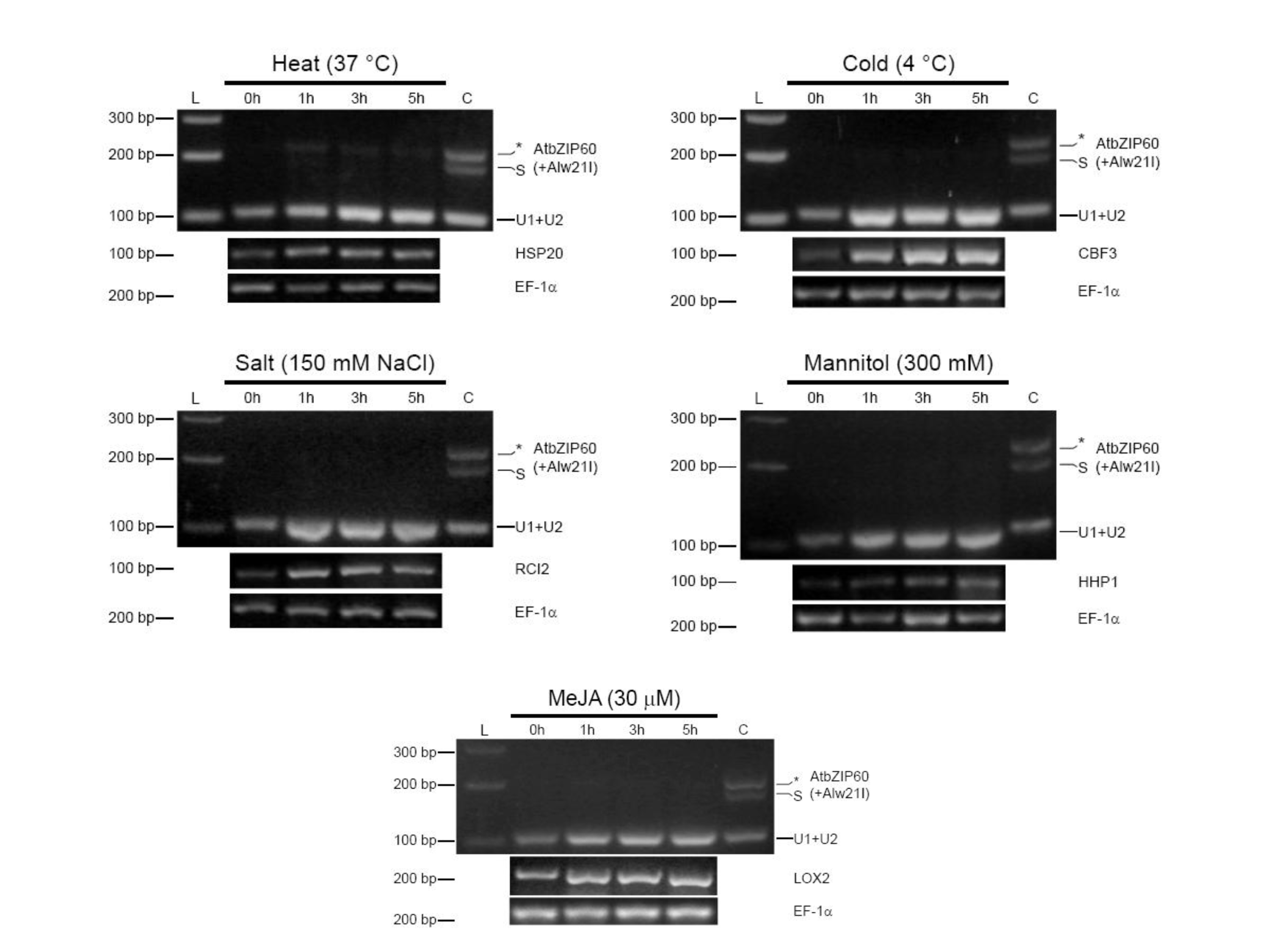

Supplement: Figure S10 — bZIP60 processing upon diverse abiotic and biotic stresses. RT-PCR products derived from bZIP60 mRNA were digested with Alw21I and resolved by gel electrophoresis in agarose (3.5% p/v). RNA samples were obtained from seedlings (6-day-old) of wild-type plants exposed to indicated treatments. C corresponds to a RNA sample obtained from seedlings treated with DTT (5 mM) for 2 hours (positive control to visualize splicing). L stands for DNA ladder. Expression levels of HSP20, CBF3, RCI2, HHP1, and LOX2 served as controls for the action of heat, cold, salt, mannitol and MeJA, respectively. Elongation factor 1 alpha (EF-1α) gene expression served as a control. (TIF) [file pone.0031944.s010.tif]

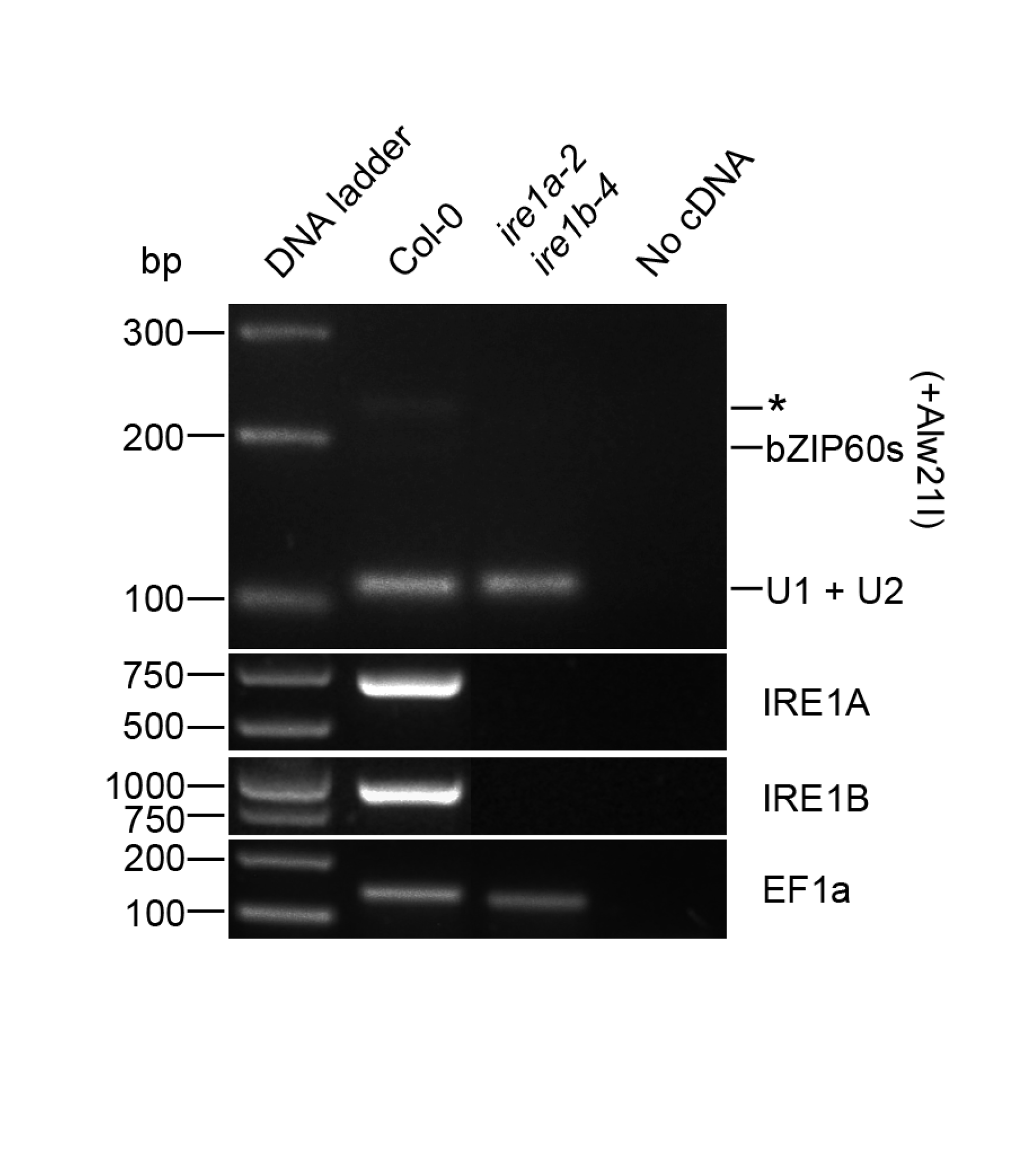

Supplement: Figure S11 — bZIP60 processing upon SA treatment in wild-type and ire1a ire1b double mutant plants. RT-PCR products derived from bZIP60 mRNA were digested with Alw21I and resolved by gel electrophoresis in agarose (3.5% p/v). RNA samples were obtained from 6-day-old seedlings treated with SA for 3 hrs. Expression levels of IRE1A and IRE1B were determined in the same samples. No cDNA was used as a negative control for background amplification. Elongation factor 1 alpha (EF-1a) gene expression served as a loading control. (TIF) [file pone.0031944.s011.tif]

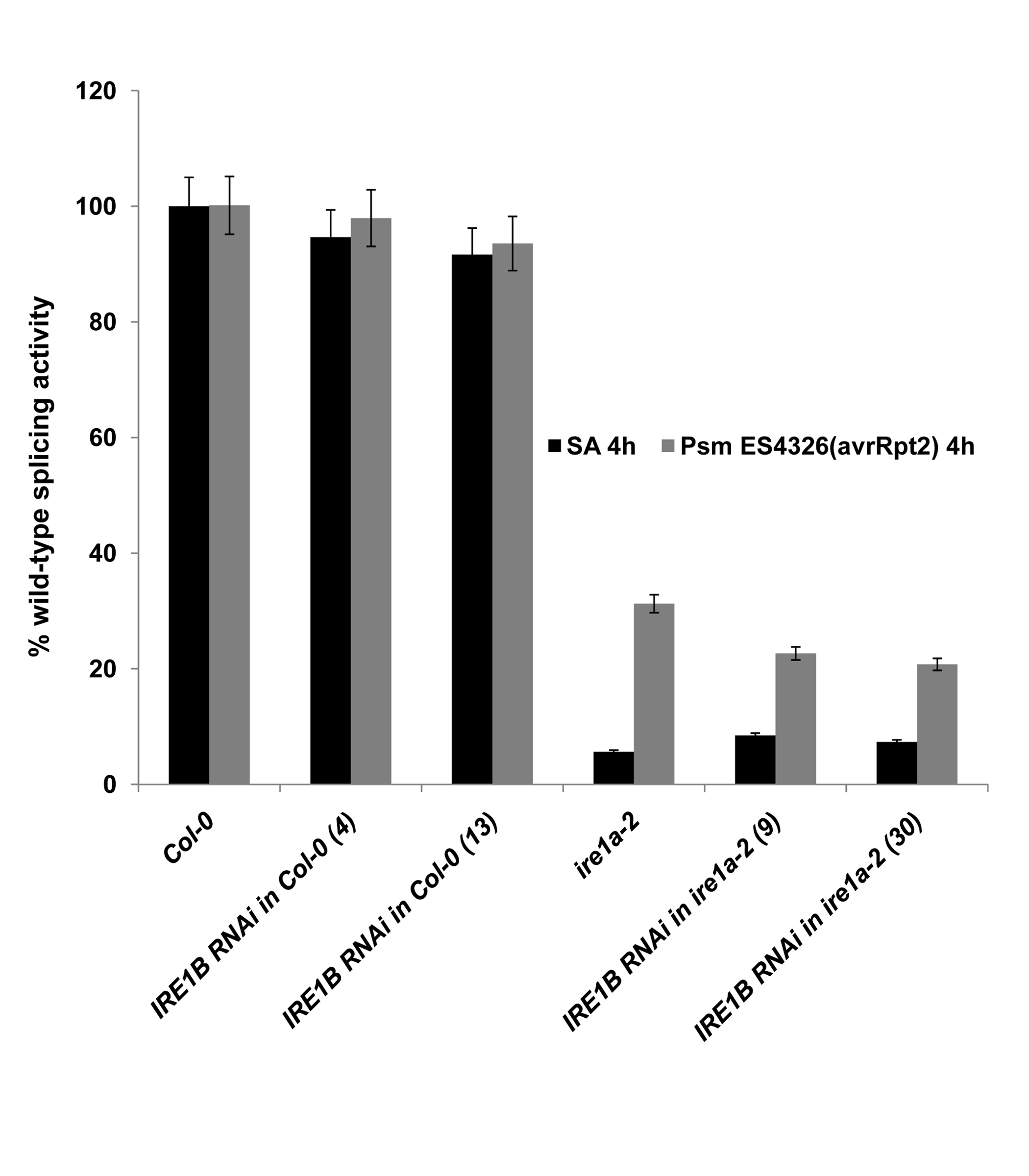

Supplement: Figure S12 — Pathogen infection- and SA-dependent bZIP60 splicing activity. cDNAs were made from the leaf tissues of the indicated genotypes, untreated or treated with Psm ES4326(avrRpt2) and SA for 4 hours. Ratios of fold induction of spliced and unspliced bZIP60 are plotted, while adjusting ratio of Col-0 as 100%. All the experiments were performed at least three times with similar results. (TIF) [file pone.0031944.s012.tif]

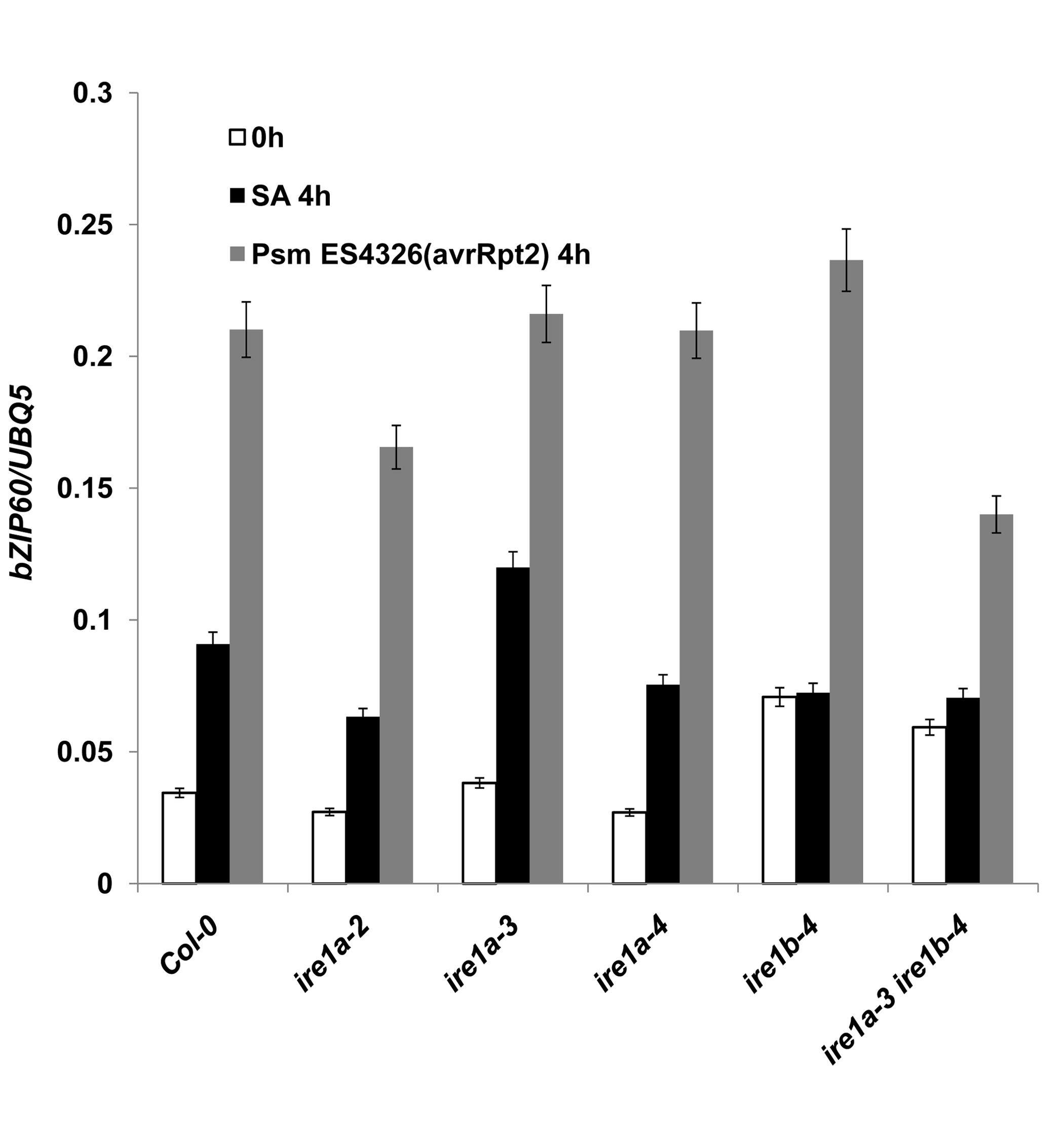

Supplement: Figure S13 — bZIP60 transcript accumulation in Col-0 and various ire1 mutants upon SA or pathogen treatment. cDNA was prepared from the leaf tissues of the indicated genotypes upon treatment with SA or PsmES4326(avrRpt2) for 4 hours as well as from untreated leaf tissues. bZIP60 transcript was measured using real-time RT-PCR. Transcript abundance was normalized using UBQ5. The experiment was performed at least three times with similar results. (TIF) [file pone.0031944.s013.tif]

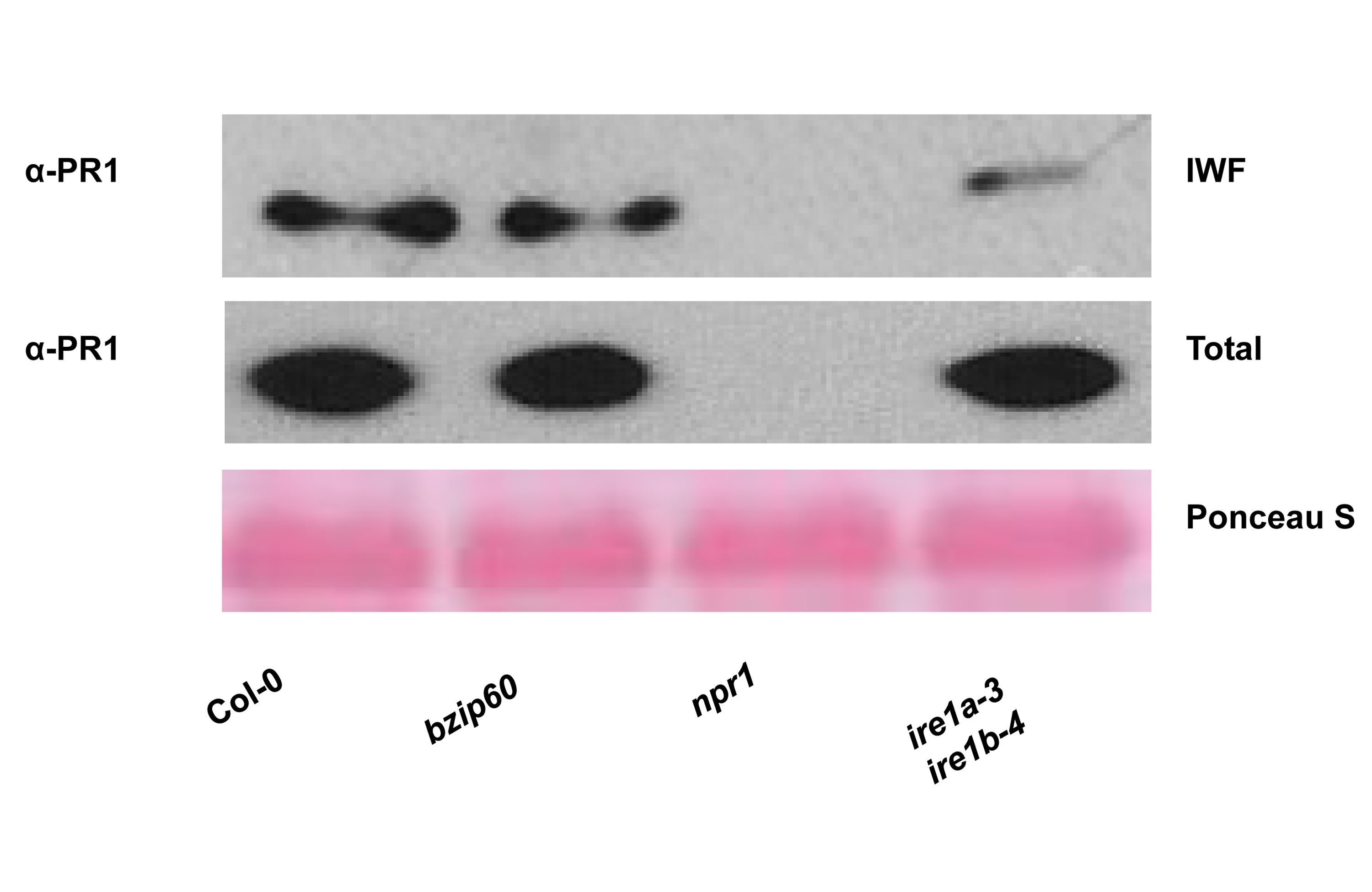

Supplement: Figure S14 — Total and secreted PR1 protein accumulation in bzip60 plants. Intercellular wash fluid (IWF) was collected from 20 leaves derived from 10 plants per indicated genotype treated with SA 16 hours prior to sampling. Total protein was extracted from five leaves derived from three plants per indicated genotype. Accumulation of PR1 was detected by Western blots with anti-PR1 antibody in IWF and total leaf extracts from the indicated genotypes. The npr1 mutant (Non-expressor of PR1) was used as control. Ponceau S stain verifies equal loading. Experiments were repeated at least four times with similar results. (TIF) [file pone.0031944.s014.tif]

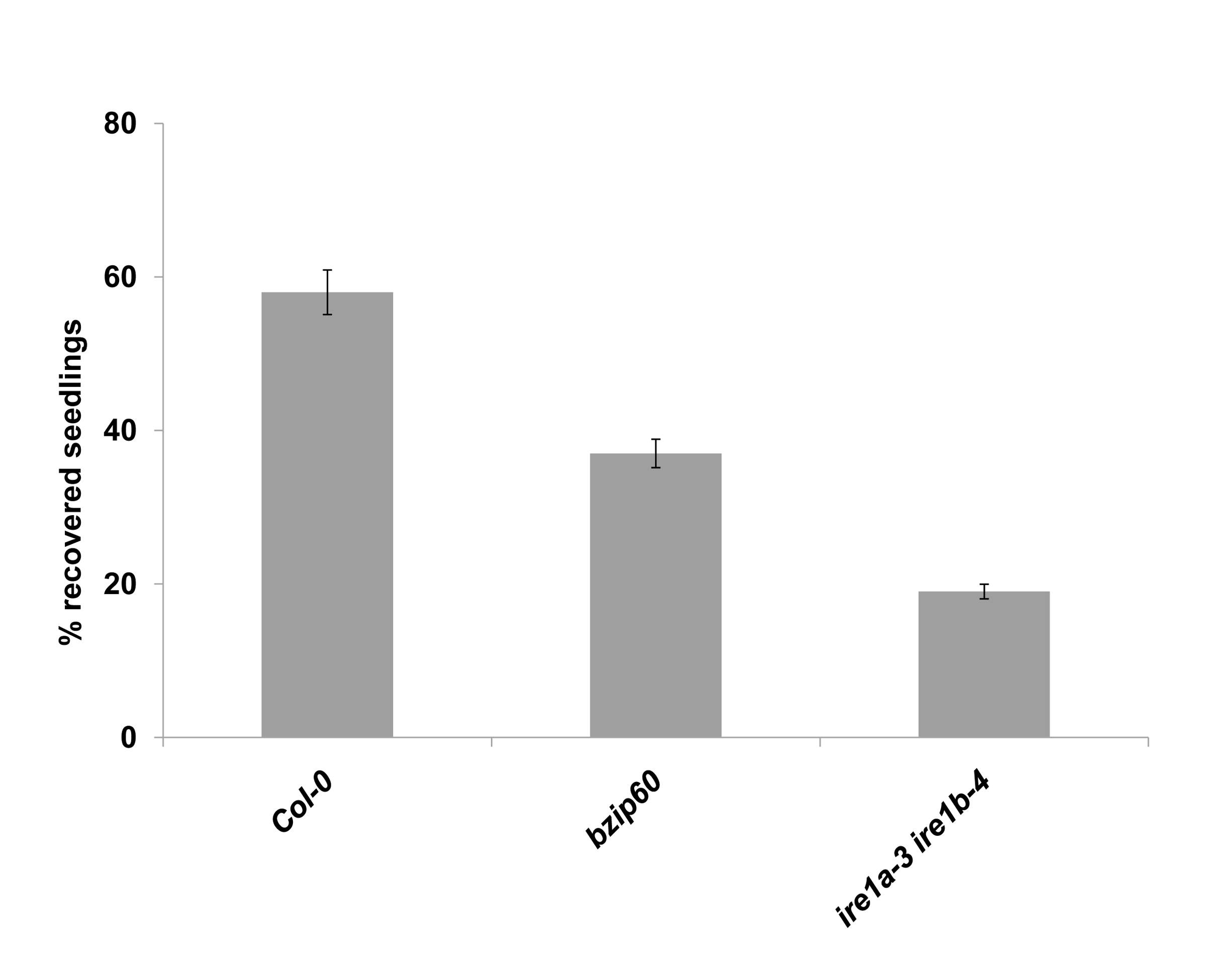

Supplement: Figure S15 — UPR stress tolerance in bzip60 seedlings. Wild-type, bzip60 and ire1a-3 ire1b-4 seedlings were grown on MS medium containing 0.3 µg/mL Tm for three days. Percentage of recovery was plotted by calculating alive/dead seedlings ten days post Tm treatment. Experiments were repeated at least three times with similar results. (TIF) [file pone.0031944.s015.tif]
