# Supplementary material for: IRE1/bZIP60-Mediated Unfolded Protein Response Plays Distinct Roles in Plant Immunity and Abiotic Stress Responses
Source: PLoS One. 2012 Feb 16;7(2):e31944. doi: 10.1371/journal.pone.0031944 (PMC3281089; doi:10.1371/journal.pone.0031944)
Supplement: Table S1 — List of primers used in this study. PCR primers used for RT-PCR, q-PCR, mutants genotyping and generation of constructs described in the manuscript are listed, alongside with the loci identifiers for the corresponding genes. (DOC) [file pone.0031944.s016.doc]

| **Primers used for RT-PCR analysis** | | | |
| --- | --- | --- | --- |
| Name | Sequence 5'->3' | Target | Locus |
| hsp20.1 | TCGTGTGGAGAGGTCGAGC | HSP20 | At1g07400 |
| hsp20.2 | GCCAGAGATATCAATAGACTTAACTTG |  |  |
| hhp1.1 | AGTGCCAGAAAGGCTTAAACCG | HHP1 | At5g20270 |
| hhp1.2 | TTAACAACCAACGTGGTCACGC |  |  |
| cbf3.1 | ATGCACGATGAGGCGATGTTTG | CBF3 | At4g25480 |
| cbf3.2 | TTAATAACTCCATAACGATACGTCGTC |  |  |
| rci2.1 | GCTACTTTCGTTGATATTATTATCGCC | RCI2 | At3g05880 |
| rci2.2 | GTGAGGACATAAATGGCGTATATGAT |  |  |
| ef1a.1 | TCACCCTTGGTGTCAAGCAGAT | EF1α | At5g60390 |
| ef1a.2 | CAGGGTTGTATCCGACCTTCTT |  |  |
| Grxc9_Rt_Fw | CCTACATAAACCGCCGGTAAC | GRXC9 | At1g28480 |
| Grxc9_Rt_Rv | GAGGCTGCTTCTTGGACTTG |  |  |
| lox2_for | CAGTTCTCATTAACAGGGATAGAT | LOX2 | At3g45140 |
| lox2_rev | CTTTAGAGCCTCATCAACTGTC |  |  |
| AMV019 | AGGACGTATGCTTGAGTGCTTCGT | bZIP60 | At1g42990 |
| AMV020 | TTCTGGACGTAGGAGGCAACACT |  |  |
| IRE1b_for | GTTAATGAGGGATATAGTTGCTG | IRE1b | At5g24360 |
| IRE1b_rev | AAGAATCCTAGAATACAGTGGTC |  |  |
| IRE1a_for | ATTGCAAAGGGAAGTAACGGA | IRE1a | At2g17520 |
| IRE1a_rev | AGATCATCACCAAAGGGATGC |  |  |
| **Primers used for q-PCR analysis** | | | |
| IRE1a_FWD | GCTTCAGACCTCATATCCCG | IRE1a | At2g17520 |
| IRE1a_REV | AGCATCACGAAGGAAAGACAG |  |  |
| IRE1b_FWD | GGTGGGATGAGAAACTGGATAG | IRE1b | At5g24360 |
| IRE1b_REV | AGTTTGTTCCGTATGACCCG |  |  |
| SRO2_FWD | TGTTCTCTACTTGCGGCTTC | SRO2 | At1g23550 |
| SRO2_REV | CACACCAGAATCAAACTCAGC |  |  |
| GLP1_FWD | TTGCTCTATCCAATGCCTCTG | GLP1 | At1g72610 |
| GLP1_REV | TGTAGTGTTTCCAGGAGTGC |  |  |
| UBQ5_FWD | GACGCTTCATCTCGTCC | UBQ5 | At3g62250 |
| UBQ5_REV | gtaaacgtaggtgagtcca |  |  |
| bZIP60us_FWD | GGAGACGATGATGCTGTGGCT | bZIP60 | At1g42990 |
| bZIP60u_REV | cagggattccaacaagagcacaG |  |  |
| bZIP60s_REV | CAGGGAACCCAACAGCAGACT |  |  |
| bZIP60_FWD | GCCTATTCCCTTATATGTCCCAC |  |  |
| bZIP60_REV | GAACCCTTACATCTCCGACTAAC |  |  |
| **Primers used for *ire1b-4* mutant genotyping**  **(WT allele: ire1b-4_for + ire1b-4_rev) (Mutant allele: ire1b-4_for + LB3)** | | | |
| ire1b-4_for | GACTAGAAACTCAACTGGTAA | IRE1b | At5g24360 |
| ire1b-4_rev | TCTTGTGCTCTCGGTCTG |  |  |
| LB3 | TAGCATCTGAATTTCATAACCAATCT  CGATACAC | Left-Border | SAIL lines |
| **Primers used for *ire1a-2* mutant genotyping**  **(WT allele: ire1a-2_for + ire1a-2_rev) (Mutant allele: ire1a-2_for + LB3)** | | | |
| ire1a-2_for | GAAAACAACGATTCTACTGAAGG | IRE1a | At2g17520 |
| ire1a-2_rev | TTGCGAGATCAATCAGTCCT | IRE1a | At2g17520 |
| LBb1.3 | ATTTTGCCGATTTCGGAAC | Left-Border | SALK lines |
| **Primers used for *ire1a-3* mutant genotyping**  **(WT allele: ire1a-3_for + ire1a-3_rev) (Mutant allele: ire1a-3_for + WiscDsLox-LP )** | | | |
| ire1a-3_for | TATCTCCGATCCATCGTTGAC | IRE1a | At2g17520 |
| ire1a-3_rev | CAAAATCTTCAGTGCTAGCGG | IRE1a | At2g17520 |
| WiscDsLox-LP | aacgtccgcaatgtgttattaagttg | Left-Border | WiscDsLox lines |
| **Primers used for *ire1a-4* mutant genotyping**  **(WT allele: ire1a-4_for + ire1a-4_rev) (Mutant allele: ire1a-4_for + LB3)** | | | |
| ire1a-4_for | GGCTACTACTGTCGATGGCTATC | IRE1a | At2g17520 |
| ire1a-4_rev | CTCCTTCAATGAGCTCGAACTG | IRE1a | At2g17520 |
| SAIL_LB1 | GCCTTTTCAGAAATGGATAAATAGCCTTGCTTCC | Left-Border | SAIL lines |
| **Primers used for amplification of the IRE1b RNAi fragment** | | | |
| Ire1b-RNAi-F | GwF-CTAAAGCCTCAAAATGTGTTGATTGT | IRE1b | At5g24360 |
| Ire1b-RNAi-R | GwR-TCAAATTTGGATCCGGGTTTAGGAG | IRE1b | At5g24360 |
